# Supplementary material for: Influenza vaccination of school teachers: A scoping review and an impact estimation
Source: PLoS One. 2022 Aug 11;17(8):e0272332. doi: 10.1371/journal.pone.0272332 (PMC9371289; doi:10.1371/journal.pone.0272332)
Supplement: S1 File — (DOCX) [file pone.0272332.s001.docx]

**S1 Supplementary. Scientific literature review**

This supplement provides the following sections:

***Methods, Results, Tables.***

***Methods***

*Search strategy.* The literature review was performed in April 2020 and was initiated by explorative searches in PubMed, Embase and Google (Scholar) to define relevant search terms. Subsequently, Embase was searched for scientific (i.e. peer-reviewed) publications. Search terms were divided into two groups relating to influenza vaccination (i.e. influenza vaccine, immunization) and to teachers (i.e. school teacher, school staff) (table S1.1, below). Search terms contained mostly one explode-term (“exp”) combined with one term present in title and/or abstract (“ti,ab”). Explode was used to include all narrower terms from Emtree. The term present in title and/or abstract was added to ensure a more confined search, to avoid a large number of mostly irrelevant hits. Search terms within each group were linked with Boolean operator ‘OR’. The groups were linked together using Boolean operator ‘AND’ leading to results including at least one of the search terms in each subgroup. All peer-reviewed English language articles published up to March 2020 with titles or abstracts describing influenza vaccination for school staff were considered potentially relevant.

*Selecting articles.* All scientific search results were screened by title and abstract by one reviewer (AH). Subsequently, full-text assessment of the potential relevant scientific search results was conducted by one reviewer (AH). Publications were excluded if they were not about influenza vaccination or not about teachers.

*Charting information and summarizing results.* From the selected relevant literature, data of interest on categories was extracted into pre-defined tables by one reviewer (AH). The categories comprised population (type of school teachers), reasons for implementing or receiving influenza vaccination, practical implementation of vaccination, vaccination coverage, reasons for not receiving influenza vaccination, possibilities to increase influenza vaccination uptake, teacher attitude towards vaccination, and the impact teacher vaccination. In addition, descriptive information was tabulated, including year of publication, country, study period and study design.

***Results***

*Characteristics.* The 12 included scientific articles about teacher influenza vaccination were published between 2007 and 2019 (table S1.2 and S1.3, below). Of these studies, 75% (n=9) was conducted in the USA, the other three were conducted in Poland (n=1), Greece (n=1) and Australia (n=1). Most studies had a questionnaire/survey-based study design (n=9, 75%), two were intervention studies, also encompassing a survey [[1](#_ENREF_1)] [[1](#_ENREF_1), [2](#_ENREF_2)] and one was a retrospective cohort study [[3](#_ENREF_3)]. Evaluating VU and assessing its determinants like knowledge and attitudes was the aim of five studies. Other aims included evaluation of an intervention on attitudes and knowledge of influenza vaccination (n=2) [[1](#_ENREF_1), [2](#_ENREF_2)], assessment of disaster readiness of schools (n=2) [[4](#_ENREF_4), [5](#_ENREF_5)], assessment of prevalence of influenza-like-illness (n=1) [[6](#_ENREF_6)], evaluation of any protective effect of ordinary seasonal vaccination against the novel pandemic H1N1 influenza by measuring teacher absenteeism pre and post school closure (n=1) [[3](#_ENREF_3)] and evaluation of acceptance to promote hygiene measures, and knowledge regarding H1N1 influenza A virus and vaccine (n=1)[[7](#_ENREF_7)]. Teacher populations differed between studies; 83% (n=10) included all three: elementary, middle and high school teachers [[1](#_ENREF_1), [4-6](#_ENREF_4), [8](#_ENREF_8), [9](#_ENREF_9)]; or one or two of these populations [[3](#_ENREF_3), [10-12](#_ENREF_10)]. Two of these studies additionally included pre-school or college teachers [[4](#_ENREF_4), [9](#_ENREF_9)] and two studies focused on nursery teachers or pre-service and early career teachers [[2](#_ENREF_2), [7](#_ENREF_7)].

*Implementation details of vaccination.* Five studies did not report the practical organisation of influenza vaccination for teachers [[6](#_ENREF_6), [7](#_ENREF_7), [9-11](#_ENREF_9)] and the other seven publications gave a limited description only (table S1.3, below). Three studies reported it being offered for free [[1](#_ENREF_1), [4](#_ENREF_4), [8](#_ENREF_8)] and two studies reported it not being free-of-cost for teachers [[2](#_ENREF_2), [12](#_ENREF_12)]. One study reported mandatory vaccination policy for school staff in 0.1% of participating schools [[4](#_ENREF_4)]. One intervention study reported on-site vaccination at schools, other studies did not report any location.

*Vaccination uptake (VU).* VU in the teacher population was mentioned in the majority of the articles (n=11) and differed by country/region and season ranging from 5-10% in European countries and from 46%-74% in Australia and USA: 2005/2006 62% (USA), 2007/2008 51% (Australia), 2008/2009 46% (USA) and 10.4% (Greece), 2009/2010 58% (Australia), 62% (USA) and 21.4% (Greece), 2010/2011 73.7% (USA), 2012/2013 55% (USA) and 58% (USA) and 2018/2019 4.7% (Poland). Three of these studies in addition compared the uptake in the studied teacher population with the general population: two find comparable percentages (4.7% vs 3.7% in Poland, 36% in the USA) [[2](#_ENREF_2), [12](#_ENREF_12)] and one study performed in the USA finds higher VU in the teacher population (55%) compared to the general adult population (42%). Three studies reported that influenza vaccination is recommended for everyone aged > 6 months in the USA and in Australia [[8](#_ENREF_8), [13](#_ENREF_13), [14](#_ENREF_14)]. The USA started recommending this in 2010 and vaccination uptake is generally higher than in Europe. It was recommended during the study periods of all but one included American study [[14](#_ENREF_14)]. In Poland, yearly influenza vaccination is recommended for those working in educational institutions [[12](#_ENREF_12)]; in Greece, influenza vaccination is not officially recommended for teachers [[15](#_ENREF_15)]. Three articles mentioned lack of easily accessible vaccination as a possible explanation for low VU [[2](#_ENREF_2), [9](#_ENREF_9), [12](#_ENREF_12)], of which one also adds cost and perceived risk as explanations [[2](#_ENREF_2)]. Facilitating immunization within school setting was mentioned most often as possibility to increase VU (n=4) [[5](#_ENREF_5), [8](#_ENREF_8), [9](#_ENREF_9), [12](#_ENREF_12)]. Other possible explanations and solutions given are correcting teacher misconception by education programs (n=3) [[2](#_ENREF_2), [11](#_ENREF_11), [12](#_ENREF_12)], encouraging workers to get vaccinated (n=3) [[5](#_ENREF_5), [6](#_ENREF_6), [8](#_ENREF_8)], reducing the costs (n=2) [[8](#_ENREF_8), [12](#_ENREF_12)], a mandatory vaccination policy (n=2) [[4](#_ENREF_4), [5](#_ENREF_5)] and understanding and addressing teachers’ attitudes (n=1) [[10](#_ENREF_10)].

*Teachers’ attitude.* The studies reported factors as teachers own health (n=4 studies), protecting those who they come into contact with (n=3) and recommendation for influenza vaccination by either the government or their doctor (n=2) as main reasons for taking the seasonal influenza vaccination. Reported motivations for not getting the seasonal influenza vaccination were lack of conviction regarding effectiveness (n=3 studies) [[8](#_ENREF_8), [9](#_ENREF_9), [12](#_ENREF_12)], concerns regarding adverse effects (n=3) [[7](#_ENREF_7), [11](#_ENREF_11), [12](#_ENREF_12)], lack of necessity due to self-perceived good health (n=3) [[8](#_ENREF_8), [9](#_ENREF_9), [11](#_ENREF_11)] and lack of time or having forgotten it (n=2) [[8](#_ENREF_8), [9](#_ENREF_9)]. Three studies reported that teachers believe they are at high risk of getting seasonal influenza or that staff and children can spread flu among each other [[2](#_ENREF_2), [8](#_ENREF_8), [12](#_ENREF_12)]. Other attitudes reported by single studies are given in table S1.3 below.

*Impact.* Only one article reported the impact of influenza vaccination on teacher absenteeism [[3](#_ENREF_3)]. It found that those vaccinated had lower absenteeism than those non-vaccinated for four months after vaccination in 2007 (0.7% absenteeism vs 3.6%, p<0.001, on a study population size of 98, Gold Coast, Queensland, Australia), but found no effect in 2009 when the vaccine viruses did not match the circulating viruses.

***Tables***

Table S1.1. Complete list of English search terms sorted by corresponding subgroup for scientific search.

| Influenza vaccination | Teachers |  |
| --- | --- | --- |
| Influenza vaccine /exp | School teacher /exp |  |
| Influenza vaccination /exp | Teacher /exp AND ‘school*’:ti,ab |  |
| ‘influenz*’:ti AND ‘vaccin*’:ti | Staff /exp AND ‘school*’:ti,ab |  |
| Influenza virus /exp | Worker /exp AND ‘school*’:ti,ab |  |
| Flu like syndrome /exp AND ‘vaccin*’:ti,ab | Employee /exp AND ‘school*’:ti,ab AND ‘teacher*’:ti,ab |  |
| Vaccination coverage /exp AND ‘influenz*:ti,ab | ‘teacher’:ti |  |
| Immunization /exp AND ‘influenz*:ti,ab | ‘schoolteacher’ |  |
| Influenza /exp AND ‘vaccin*’:ti,ab |  |  |

The asterisk (*) was used as a wildcard to allow end-truncation. Front truncation was not available.

Table S1.2. Overview of relevant scientific publications and their aims

| First author, publication year | Journal | Study period | Location | Aim | Study design |
| --- | --- | --- | --- | --- | --- |
| Ganczak M,  2019 [[12](#_ENREF_12)] | Vaccine | March to May 2019 | Poland | To evaluate influenza vaccination coverage and to assess influencing determinants among Polish teachers. | Cross-sectional questionnaire-based study |
| Vaughn AR,  2018 [[2](#_ENREF_2)] | Vaccine | Not mentioned | USA | To assess the effectiveness of a refutational text on pre-service and early career teachers’ attitudes toward and knowledge of influenza and influenza vaccination. | Intervention and questionnaire / survey |
| Rebmann T,  2015[[5](#_ENREF_5)] | American Journal of Infection Control | 2013-2014 school year | Missouri, USA | To conduct an updated, more thorough assessment of Missouri ‘K-12’ schools’ readiness for disasters, particularly for large-scale biological events, including emerging infectious diseases, bioterrorism, and pandemics. | Survey |
| De Perio MA,  2014 [[6](#_ENREF_6)] | Journal of School Health | March 2013 | Ohio, USA | To determine 2012-2013 influenza vaccination coverage among employees in the school district, assess employees’ knowledge and attitudes toward vaccination, and determine factors associated with acceptance and refusal of the vaccine. | Cross-sectional study; survey |
| Macintosh J,  2014 [[9](#_ENREF_9)] | Vaccine | Not mentioned | Utah, USA | To evaluate why school employees have not received the adult MMR vaccine or an annual influenza vaccine and to identify views on vaccination mandates for school employees in a rural school district in Utah. | Questionnaire for both quantitative and qualitative analysis |
| De Perio MA,  2014 [[8](#_ENREF_8)] | American Journal of Infection Control | March 2013 | Ohio, USA | To determine the prevalence of influenza-like illness among employees of a suburban Ohio school district. | Cross-sectional study; survey |
| Rebmann T,  2012 [[4](#_ENREF_4)] | American Journal of Infection Control | May to July 2011 | USA | To evaluate US schools’ and academic institutions’ current state of readiness to respond to a disaster, particularly focusing on preparedness for infectious disease disasters, such as bioterrorism, pandemics, and outbreaks of emerging infectious diseases. | Survey |
| Yin JK,  2011 [[3](#_ENREF_3)] | Australian and New Zealand Journal of Public Health | May to October in 2007 and 2009 | Queensland, Australia | To assess the cross-protection against 2009 pandemic H1N1 influenza due to seasonal vaccination and explore the effect of school closure on absenteeism (sick leave) of school teachers. | Retrospective cohort study |
| Gargano LM,  2011 [[10](#_ENREF_10)] | Journal of School Health | September 2009 | Georgia, USA | To assess the knowledge and attitudes of 6-12th grade teachers and staff regarding 2009 H1N1 influenza virus and the 2009 H1N1 influenza vaccine in 2 rural counties in the state of Georgia. | Survey |
| Gargano LM,  2011[[11](#_ENREF_11)] | Human Vaccines | September 2009 and May 2010 | Georgia, USA | To investigate factors associated with seasonal and 2009 H1N1 influenza vaccine uptake among middle- and high-school teachers in a rural, low-income setting during the 2009-2010 influenza season. | Survey |
| Ioannidou C,  2010[[7](#_ENREF_7)] | Abstract of a poster presentation printed by Foundation Acta Paediatrica (conference name not available) | February to March 2010 | Greece | To evaluate the acceptance of Greek nursery teachers to promote the hygiene interventions, proposed by the Hellenic CDC during the novel H1N1 influenza A epidemic. A secondary objective was to assess their knowledge regarding the novel virus and the vaccine. | Questionnaire |
| Carpenter LR,  2007 [[1](#_ENREF_1)] | Pediatrics | June to December 2005 | Tennessee, USA | To evaluate a campaign to vaccinate students with live attenuated influenza vaccine in a large metropolitan, public school system, to assess feasibility and success. | Intervention and survey |

Table S1.3. Study characteristics of the selected scientific publication

| First author, year | Country | Study design | Study period | Population (type of school teachers) | Reasons for (implementing/ receiving) vaccination | Implementation details of vaccination (distribution, costs and encouragement) | Vaccination coverage | Explanation for poor uptake + possibilities to increase uptake | Main reasons for not receiving influenza vaccination | Attitude teachers | Impact | Reported conclusion |
| --- | --- | --- | --- | --- | --- | --- | --- | --- | --- | --- | --- | --- |
| Ganczak M,  2019 [[12](#_ENREF_12)] | Poland (Szczecin and Lublin) | Cross-sectional questionnaire-based | March to May 2019 | 277 teachers from 9 primary schools | The authors state that those coming into contact with teachers require protection against influenza. | Working age adult patients are not offered insurance coverage for influenza vaccine. Not free of charge. | 4.7% in the 2018/2019 season (compared to 3.7% in 2017/2018 season in general population); 24.5% reported ever receiving it | Lack of free on-site influenza vaccine at work; include influenza vaccine within private insurance packages and provision of influenza education programs and facilitate immunization within school setting | Lack of conviction regarding effectiveness (57%); concerns regarding adverse effects (31%) | 44% believed they are at high risk; 51% were not willing to vaccinate themselves; 26% believed influenza vaccination should be mandatory for teachers | - | Influenza vaccination coverage among Polish teachers is alarmingly low; vaccine receipt was associated with teacher attitudes and beliefs. Future interventions related to maximizing vaccination coverage in this group should take advantage of the involvement of family physicians and specifically focus on teachers who have never been vaccinated before. As knowledge about influenza positively influences vaccination decisions, education strategies should focus on reducing knowledge gaps to alter attitudes and increase uptake. |
| Vaughn AR,  2018[[2](#_ENREF_2)] | USA | Intervention + survey / questionnaire | Not mentioned | 64 pre-service teachers or early career teachers | Everyone is at risk of catching the flu and CDC recommends everyone age 6 months and older to be vaccinated. Prevent your own illness and also protect those around you. | Not free of charge. | 83% reported ever receiving it; 36% reported receiving it 5 times in the prior five years (similar to general public) | Lack of easily accessible vaccination, cost or perceived risk; correct misconception and increase positive attitude | - | 89% agrees to being at risk of catching the flu; 92% agrees that it is important for teachers to be vaccinated against influenza; 91% agrees it is important for oneself to be vaccinated against influenza | - | Our findings reveal teachers have similar vaccination rates as the general public and moderate knowledge of influenza. We found refutational texts are effective in eliciting more positive attitudes toward influenza vaccination. We found cognitive engagement was positively correlated to change in attitude. Refutational texts provide an effective mode of influenza and vaccination education. |
| Rebmann T,  2015 [[5](#_ENREF_5)] | USA (Missouri) | Survey | 2013-2014 school year | 65 elementary schools, 30 middle schools and 37 high schools | Protect not only the school staff from disease, but also reduce sick leave and minimize disease spread even in nonhospital settings. | 85.7% of the schools encouraged teachers or teaching assistants to receive vaccine, but not mandatory. 1.5% of schools mandated vaccination of school nurses. | - | Having a mandatory vaccination policy and encouraging workers to get vaccinated and host a school-based immunization clinic. | - | - | - | Schools are underprepared for biological events and are not on track to meet state and national biological preparedness goals. Immunization of school staff against seasonal influenza and other vaccine preventable diseases should be a priority for school districts. |
| De Perio MA,  2014[[8](#_ENREF_8)] | USA (Ohio) | Cross-sectional study; survey | March 2013 | 5 elementary schools, 1 middle school and 2 high schools; 841 full-time and part-time employees; 412 (49%) completed the survey. | To protect oneself or one’s family (87%); “I’ve read/heard that getting flu vaccine is recommended (5%); “My doctor recommended that I get the flu vaccine”(4%) | Free of charge. Not mandatory. | 55% of teachers (and 58% of all school employees) reported getting the 2012/2013 season vaccination (compared to 42% in general US adult population) | Encourage employees to get the influenza vaccination and explore the feasibility of offering on-site annual influenza vaccination to employees at no or low cost | “I don’t think I need the vaccine” (32%); “I don’t think the flu vaccine will keep me from getting the flu”(21%); “ I haven’t had the time to get the flu vaccine” (17%) | 99% agrees teachers/staff and children can spread flu among each other; 96% agrees flu is a serious infection; 72% agrees flu vaccine will prevent them from getting sick; 59% agrees flu vaccine could make them sick. | - | Influenza vaccine coverage among school employees should be improved. Messages encouraging school employees to get the vaccine should address misconceptions about the vaccine. Employers should use methods to maximize employee vaccination as part of a comprehensive influenza prevention program. |
| Macintosh J,  2014 [[9](#_ENREF_9)] | USA (Utah) | Questionnaire for both quantitative and qualitative analysis | Not mentioned | Pre-school, elementary, junior high school, high school, district office building; 1346 employees of which 835 responded (62%) | - | - | 49% of all school employees reported receiving vaccination in 2011/2012 season | Inconvenience of seeking and receiving vaccination; school located vaccination clinics | “Do not believe immunizations would help” (13.4%); “Did not have time/forgot”(12.9%); “I am healthy and do not need it” (10.9%) |  | - | Suboptimal vaccination rates of school employees may negatively affect the health and well-being of individuals in the school environment. School employees report a variety of beliefs regarding the influenza and MMR vaccines. While over half of school employees support mandatory vaccination policies for adults working in the school environment, those opposing such a policy report concerns regarding violation of personal choice. Public health officials and school administrators should coordinate efforts to increase vaccination rates among adults in the school environment. |
| De Perio MA,  2014 [[6](#_ENREF_6)] | USA (Ohio) | Cross-sectional study; survey | March 2013 | 5 elementary schools, 1 middle school and 2 high schools; 841 employees of which 412 (49%) completed the survey. | - | - | 238 respondents (58%) received vaccination in 2012/2013 season | Encouragement to receive the seasonal influenza vaccination | - | - | - | The prevalence of ILI among responding school employees was 29%, and 77% reported working while ill. Encouraging school employees to receive the influenza vaccine and to stay home when ill should be part of a comprehensive influenza prevention strategy in school districts. |
| Rebmann T,  2012 [[4](#_ENREF_4)] | USA (multiple states) | Survey | May to July 2011 | 1,997 school nurses (21.9%) from 26 states completed the survey; elementary school (41.3%), high school (36.5%), middle school (21.0%), college or university (1.2%). | - | 949 (64.5%) reported receiving the vaccine free of charge; 43 (2.2%) reported that their school had a mandatory influenza vaccination policy for nurses; 1 (0.1%) reported mandatory influenza vaccination policy for other school staff; 1,609 (80.6%) reported being encouraged by their employer to receive influenza vaccine. | 1,472 (73.7%) reported receiving vaccination in 2010/2011 season | Mandatory vaccination policies | - | - | - | School preparedness for disasters and infectious disease emergencies is essential, yet many schools are lacking in adequate plans.  Among several other conclusions about disaster preparedness in general, this paper reports: School nurse seasonal vaccine compliance will facilitate vaccine compliance during a disaster or pandemic, which should decrease event-related morbidity and mortality. |
| Yin JK,  2011 [[3](#_ENREF_3)] | Australia (Queensland) | Retrospective cohort study | May to October in 2007 and 2009 | Teachers at a senior school | - | Routinely offered to teachers. | Season 2007 50/98 (51%) of the teachers was vaccinated. Season 2009 58/99 (58%) of the teachers was vaccinated. | - | - | - | With days absent (sick leave) per month in staff as outcome: after the school holiday in July 2007, those vaccinated had much lower absenteeism than those non-vaccinated (0.7% vs 3.6%, p<0.001; RR was 0.18 (95% CI: 0.006-0.53). Higher overall absence rates in July 2009 were reported. No significant difference between vaccinated (5.9%) and non-vaccinated (6.8%) was found. | Our data does not support a protective effect of 2009 TIV against pandemic H1N1 influenza but a systematic review of published studies is underway. However, we showed that seasonal vaccination of teachers in 2007 appeared beneficial, probably because there was a close match with the seasonal strain. |
| Gargano LM,  2011[[10](#_ENREF_10)] | USA (Georgia) | Survey | September 2009 | A single middle and high school from 2 rural counties; 102/161 (63%) school staff completed and returned the baseline survey | - | - | In 2008/2009 season 46% received seasonal influenza vaccination; 68% reported ever receiving it in the past. | Understanding and addressing  teachers’ attitudes toward H1N1 vaccination | - | - | - | Teachers may play a pivotal role in school-based H1N1 vaccinations. Understanding and addressing teachers’ attitudes toward H1N1 vaccination may assist in future immunization efforts. |
| Gargano LM,  2011 [[11](#_ENREF_11)] | USA (Georgia) | Survey | September 2009 and May 2010 | Middle- and high-schools in two rural counties; follow-up survey was completed by 66/161 (41%) school staff members (of which 88% were teachers); 57 (86%) of those also completed baseline survey | Wanting to avoid getting seasonal influenza (93%); knowing someone who got sick from seasonal influenza (81%); hearing about seasonal influenza on the news (78%) | - | In 2009/2010 season 41 (62%) reported receiving seasonal influenza vaccination | Influenza vaccination programs which focus on safety, risk and social norms (take into account psychosocial factors to the pandemic vaccine). | Concern the seasonal influenza vaccine would make them sick (64%); the belief that they did not need the seasonal influenza vaccine (56%); concern about side effects (52%) | Participants with higher perceived severity of seasonal influenza and increased self-efficacy for seasonal influenza vaccination demonstrated increased odds of receiving a seasonal influenza vaccination. | - | There is a strong association between the intention to be vaccinated against influenza (seasonal or 2009 H1N1) and actual vaccination uptake. Understanding and addressing factors associated with teachers’ influenza vaccine uptake may enhance future influenza immunization efforts. |
| Ioannidou C,  2010 [[7](#_ENREF_7)] | Greece | Questionnaire | February to March 2010 | 557 nursery teachers (92.8% response rate) | - | - | 10.4% during 2008-09 season and 21.4% during 2009-10 season reported uptake of seasonal influenza vaccine. 1.1% reported receiving the pandemic influenza vaccine. | - | For the pandemic vaccine: fear of adverse reactions (55.7%). | - | - | Simple preventive (hygiene red.) measures were promoted effectively by more experienced teachers and better adapted by older children during the novel influenza threat. Knowledge gaps and distrust to the pandemic vaccine, highlight communication issues. |
| Carpenter LR,  2007 [[1](#_ENREF_1)] | USA (Tennessee) | Intervention + survey | June to December 2005 | 50 elementary schools, 14 middle schools and 12 high schools | - | Live attenuated influenza vaccine; free of charge | 3626/5841 (62%) school staff members were vaccinated (40% LAIV and 60% TIV). The staff vaccination level in elementary  schools was 68%, middle schools 64%, and high schools 58%. | - | - | - | - | This influenza vaccination campaign in a large public school system achieved relatively high vaccine coverage levels but required a substantial resource commitment from the local health department. This evaluation has critical implications for the ongoing debate regarding immunization policies for school-aged children and preparedness plans for pandemic influenza. (the authors assumed that strategies for mass vaccination have implications for planning for pandemic influenza) |

***References***

1. Carpenter L. Mass distribution of free, intranasally administered influenza vaccine in a public school system. Pediatrics. 2007;120(1).

2. Vaughn A. Communicating and enhancing teachers attitudes and understanding of influenza using refutational text. Vaccine. 2018;36(48):7306-15.

3. Yin J. Assessing seasonal vaccine-related cross-protection from 2009 pandemic H1N1 influenza through teacher absenteeism. Australian and New Zealand journal of public health. 2011;35(4):393-4.

4. Rebmann T. U.S. school/academic institution disaster and pandemic preparedness and seasonal influenza vaccination among school nurses. American Journal of Infection Control. 2012;40(5):45.

5. Rebmann T. Missouri K-12 school disaster and biological event preparedness and seasonal influenza vaccination among school nurses. American Journal of Infection Control. 2015;43(10):1028-34.

6. de Perio M. Influenza-like illness and presenteeism among school employees. American Journal of Infection Control. 2014;42(4):450-2.

7. Ioannidou C. Promotion of hygiene measures to prevent pandemic influenza transmission in greek nursery schools: The teachers'a perspectives. Acta Paediatrica. 2010;99:88.

8. de Perio M. Influenza vaccination coverage among school employees: assessing knowledge, attitudes, and behaviors. Journal of School Health. 2014;84(9):586-92.

9. Macintosh J. Vaccination perceptions of school employees in a rural school district. Vaccine. 2014;32(37):4766-71.

10. Gargano L. Correlates of 2009 pandemic H1N1 influenza vaccine acceptance among middle and high school teachers in rural Georgia. Journal of School Health. 2011;81(6):297-303.

11. Gargano L. Seasonal and 2009 H1N1 influenza vaccine uptake, predictors of vaccination and self-reported barriers to vaccination among secondary school teachers and staff. Human Vaccines. 2011;7(1):89-95.

12. Ganczak M. School life and influenza immunization: A cross-sectional study on vaccination coverage and influencing determinants among Polish teachers. Vaccine. 2019.

13. Clinical update: 2020 seasonal influenza vaccines - early advice for vaccination providers: Australian Government; [updated 2 March 2020]. Available from: <https://www.health.gov.au/news/clinical-update-2020-seasonal-influenza-vaccines-early-advice-for-vaccination-providers>.

14. Armstrong C. CDC updates guidelines for influenza vaccination for 2010-2011 season. Am Fam Physician. 2010;82(7):838-44.

15. Seasonal influenza vaccination and antiviral use in EU/EEA Member States. Stockholm: European Centre for Disease Prevention and Control, 2018.
