# Supplementary material for: Influenza vaccination of school teachers: A scoping review and an impact estimation
Source: PLoS One. 2022 Aug 11;17(8):e0272332. doi: 10.1371/journal.pone.0272332 (PMC9371289; doi:10.1371/journal.pone.0272332)
Supplement: S2 File — (DOCX) [file pone.0272332.s002.docx]

**S2 Supplementary. Grey literature review**

This supplement provides the following sections:

***Methods, Results, Tables.***

***Detailed methods***

*Search strategy.* The grey literature search was restricted to English and Dutch publications published up to March 2020. Defined Dutch search terms were categorized into three subgroups (table S2.1, below). Within each subgroup terms were combined using Boolean operator “OR” and subgroups as a whole were combined with Boolean operator “AND”. The English search terms were ordered differently in the grey search than in the scientific search, to manage the limited number of advanced search functions on Google and to narrow down the number of irrelevant hits (table S2.2, below). These were combined in the same manner as the Dutch search terms. A targeted internet search resulted in many irrelevant hits, therefore the search was restricted to publications from the World Health Organization (WHO), European Centre for Disease Prevention and Control (ECDC) and Dutch organizations in the field of public health and vaccinations: the National Institute for Public Health and the Environment, two Dutch public health services (PHS) (Amsterdam and Rotterdam), Nivel (Netherlands Institute for Health Services Research), and the Health Council of the Netherlands. Each search was restricted to the domain of each of the selected organizations. In addition to the searches in the defined domains, one additional search was performed to identify the most important news articles on the subject by searching for the terms in the title only. Only reports published up to March 2020 were selected. A broad range of publication types was explored (i.e. government reports, news items).

*Selecting articles.* All search results were screened by title and abstract. Subsequently, full-text assessment of potential relevant scientific and grey search results was conducted. Publications were excluded if they were not about influenza vaccination or not about teachers.

*Charting information and summarizing results.* From the selected relevant literature, data of interest on categories was extracted into pre-defined tables by one reviewer (AH). The categories compromised publication type, population (type of school teachers), reasons for implementing or receiving influenza vaccination, practical implementation of vaccination, vaccination coverage, reasons for not receiving influenza vaccination, and the impact teacher vaccination.

***Results***

*Characteristics.* The 23 included grey publications were published or updated between March 2007 and February 2020 (table S2.3, below) [[1-23](#_ENREF_1)]. Those included were mainly Dutch language news articles (n=14, 61%), other articles were published by the Health Council of the Netherlands (n=4), ECDC (n=3), municipal health centre Amsterdam (n=1) and National Institute for Public Health and the Environment (n=1). No relevant results were found from the municipal health centre Rotterdam, Nivel and World Health Organization. Publication types included news articles (n=14), reports (n=4), request for or offer of advice (n=3), Health Council work program (n=1) and an influenza vaccination information webpage (n=1). The majority of the publications specifically discussed influenza vaccination for school teachers or staff (n=17, 74%) [[1-17](#_ENREF_1)]. Other publications referred to occupational groups with intensive contacts with the population, with teachers as an example (n=5) [[18-22](#_ENREF_18)]. One publication did not specify further than employees [[23](#_ENREF_23)].

*Implementation details of vaccination.* The Council of the Netherlands did not advise vaccination of occupational groups with intensive population contacts such as teachers [[20](#_ENREF_20)]. This was based on lack of evidence that teachers would be at increased risk of complications from influenza in the event that they should fall ill. Nor are they more likely to transmit influenza to people for whom this would pose a serious threat [[20-22](#_ENREF_20)]. The Health Council additionally described that it is going to revise its advice for employees in the work program for 2020 [[23](#_ENREF_23)]. Despite this advice, some Dutch initiatives were described with voluntary uptake: the municipality of Amsterdam (2018-2020) and a schoolboard in the eastern part of the country offered the influenza vaccination free of charge, mainly motivated by increasing teacher shortages [[6](#_ENREF_6), [8](#_ENREF_8), [11](#_ENREF_11), [15-17](#_ENREF_15)]. A schoolboard across the country (8 of all 642 Dutch high schools) offered teachers a bonus for getting vaccinated (at the PHS, 405 euro bonus vs roughly 25 euro cost) [[9](#_ENREF_9), [10](#_ENREF_10), [12](#_ENREF_12)]. Vaccination uptake is voluntary in these programs. A news item from Belgium reported that schools can offer vaccination of their teachers, but that it is not often done because of the costs for schools [[4](#_ENREF_4)]. ECDC reported five EU/EEA Member States that recommend vaccination of all educational staff (Luxembourg, Poland, Slovenia) or educational staff at kindergarten and childcare facilities (Portugal, Liechtenstein) [[5](#_ENREF_5)], with employer and national health service funding as predominant payment mechanism. The vaccinations were never mandatory.

*Vaccination uptake.* Information on influenza VU in the teacher populations was limited. The vaccination campaign in Amsterdam had a VU of 2.3% in season 2018/2019 and 2.6% in season 2019/2020 [[6](#_ENREF_6), [17](#_ENREF_17)], but the campaign may not have been known among many teachers (see interview results). The schoolboard that offered a financial reward (405 euro per person) for vaccination (to be obtained at the PHS) reported a much higher VU of 69% overall and 84% specifically for their location in Kapelle, Zeeland (season 2018/2019) [[12](#_ENREF_12), [13](#_ENREF_13)].

*Attitude and impact.* Little is described on attitude and impact. A news article on the website of a political party (Christian Democratic Appeal) in The Hague called for the municipality to start offering all teachers in The Hague a free influenza vaccination [[14](#_ENREF_14)]. This and other Dutch language news articles mainly reported reducing absenteeism as motivation to vaccinate teachers for influenza (n=14, 61%). One additionally mentioned a high influenza risk for teachers [[11](#_ENREF_11)]. The National Institute for Public Health and the Environment information page described that vaccination of employees in child care and education protects them against influenza which may lead to reduced absenteeism [[18](#_ENREF_18)]. On impact, one news article stated that the number needed to vaccinate to prevent one case in healthy adults is 75 [[4](#_ENREF_4)] and one news article stated offering influenza vaccination to teachers to be less costly than hiring substitute teachers [[1](#_ENREF_1)].

***Tables***

Table S2.1. Complete list of Dutch search terms sorted by corresponding subgroup for grey search.

| Influenza | Vaccination | Teachers |
| --- | --- | --- |
| Griep | Vaccinatie | Leraren |
| Influenza | Griepprik | Docenten |
| Seizoensgriep | Griepvaccinatie | Werknemers onderwijs |
|  | Prik | Leerkrachten |
|  | Vaccin | Juffen / meesters |
|  |  | Bassisschoolleraar / basisschoolleraren |
|  |  | Schooldocent |

Table S2.2. Complete list of English search terms sorted by corresponding subgroup for grey search.

| Influenza | Vaccination | Teachers |
| --- | --- | --- |
| Influenza | Vaccination | Teachers |
| Seasonal influenza | Vaccine | School teachers |
| Flu | Immunization | School staff |
| Influenza virus |  | School employees |
| Influenza-like-illness |  | Schoolteachers |
| Flu like syndrome |  |  |

Table S2.3. Characteristics of publications in grey literature

| First author/ organization, year | Country | Publication type | Population | Reasons for (implementing/receiving) influenza vaccination | Implementation details of vaccination (distribution, costs and encouragement) | Vaccination uptake | Main reasons for not receiving influenza vaccination | Impact | | Main message about influenza vaccination for teachers |
| --- | --- | --- | --- | --- | --- | --- | --- | --- | --- | --- |
| Municipality health centre Amsterdam, 2018 [[6](#_ENREF_6)] | Netherlands | Annual report municipality health centre Amsterdam | Primary school and high school teachers;  > 9000 | - | Free of charge; at municipality health centre | 211 teachers (2.3%) in influenza season 2018-19 | - | - | “The department of Education, Youth and Care of the municipality of Amsterdam has given us the assignment to invite more than 9,000 primary and secondary school teachers to get the influenza vaccination. 211 teachers received the vaccination (2.3%).” | |
| Ministry of Health, Welfare and Sport, 2018[[22](#_ENREF_22)] | Netherlands | Request for advice on influenza to Health Council of the Netherlands | Occupational groups in which it is desirable to actively offer influenza vaccination (because of own vulnerability and aspects of the occupation) | - | - | - | - | - | “There are also groups that do not fall within the National Flu Prevention Program, but for who the active offering of influenza vaccination of influenza vaccination is recommended. An example are health care workers, who were also mentioned in the letter to the House of Representatives. I would like to hear from your committee whether there are other professional groups, besides health care providers, for who it is desirable to offer influenza vaccination. This may involve both own vulnerability and aspects of work that lead to influenza vaccination. | |
| Health Council of the Netherlands, 2007 [[20](#_ENREF_20)] | Netherlands | Advice offer on flu vaccination to minister of Health, Welfare and Sport | Occupational groups with intensive contacts with the population (like teachers) | - | - | - | - | - | “Based on available literature, there is no reason to assume that professional groups with intensive contact with the population have an increased risk of influenza or of serious illness or death from influenza. The committee also sees no reason to assume that this group has increased chance to infect people who belong to a risk group. Based in this information, the committee sees no reason to add this group to the target groups for influenza vaccination.” | |
| Health Council of the Netherlands, 2011 [[21](#_ENREF_21)] | Netherlands | Advisory letter on flu vaccination to minister of Health, Welfare and Sport | Occupational groups with intensive contacts with the population (like teachers) | - | - | - | - | - | “The council did not find arguments present for vaccination of professional groups with intensive contacts in the population, like teachers.”  “I asked the standing committee on Infection and immunity if it saw any reasons to deviate from the latest Health Council advice on influenza vaccination. Together with the standing committee I conclude that there is currently no reason for this.” | |
| Health Council of the Netherlands, 2019 [[23](#_ENREF_23)] | Netherlands | Work program 2020 Health Council of the Netherlands | Employees | - | - | - | - | - | The ministry of Health, Welfare and Sport has established a work agenda for 2018 to 2021, based on criteria for prioritization developed by the Health Council. In accordance with this agenda, the council will in 2020 work on advice on influenza vaccination […].” “Vaccination of employees against infectious diseases will also receive attention.” | |
| National Institute for Health and Environment, 2020 [[18](#_ENREF_18)] | Netherlands | Annual flu vaccination – information page | Employees in non-healthcare institutions (like daycare or education) | - | - | - | - | - | “Vaccination of employees in childcare and education protects them against flu, which results in less absenteeism. The Health Council is currently preparing an advice whether all children should also be vaccinated against flu annually. This is already the policy in some countries.” | |
| European Centre for Disease Prevention and Control, 2018 [[5](#_ENREF_5)] | Europe | Technical report Seasonal influenza vaccination  and antiviral use in  EU/EEA Member States | Teachers and other educational staff | - | Payment mechanism:  Portugal: national health service  Poland: private insurance + national health service  Liechtenstein and Slovenia: employer | - | - | - | Five EU/EEA Member States recommended the vaccination of teachers and other educational staff. Influenza season 2017-18.  Luxembourg, Poland and Slovenia: all educational staff  Portugal and Liechtenstein: educational staff at Kindergarten and crèches/child care. | |
| European Centre for Disease Prevention and Control, 2017 [[3](#_ENREF_3)] | Europe | Technical report Seasonal influenza  vaccination in Europe | Teachers and other educational staff | - | The predominant payment  mechanism for vaccination was through the employer. | - | - | - | Four Member States recommended the  vaccination of teachers and other educational staff in 2014–15 (five in 2010–11; three in 2011-12 and 2012-13).(country names not given) | |
| European Centre for Disease Prevention and Control, 2016 [[2](#_ENREF_2)] | Europe | Technical report Seasonal influenza vaccination and antiviral use in Europe | Teachers and other educational staff | - | The predominant payment mechanism for vaccination was through the employer and a  combination of several payment mechanisms | - | Expected fundamental and practical objections | - | Four Member States recommended the vaccination of teachers and other educational staff. Influenza season 2014-15.  All teachers (primary/secondary schools, preschool centres, nursery schools, crèches/child care) and administrative workers: Poland  Staff at nursery schools and crèches/child care: Liechtenstein  Other: Austria, Estonia | |
| Het Parool, 2018 [[8](#_ENREF_8)] | Netherlands | News article | Teachers of primary schools and high schools, and vocational schools | Reduce absenteeism | Voluntarily; free of charge (paid by municipality: 50,000 euros) | - | - | - | “Amsterdam is using new weapon against the teacher shortage: a free flu shot for teachers.” | |
| Het Parool, 2019 [[16](#_ENREF_16)] | Netherlands | News article | Teachers of primary schools and high schools | Reduce absenteeism | Voluntarily; free of charge (paid by municipality: 50,000 euros) | Previous year: 200 of almost 10,000 teachers | - | - | “For the second year in a row, teachers in Amsterdam can get a free flue shot. By doing this, education councilor Marjolein moorman wants to limit the effects of the seasonal flu on schools, so that fewer students are sent home or classes are merged.” | |
| Het Parool, 2019[[17](#_ENREF_17)] | Netherlands | News article | Teachers of primary schools and high schools | Reduce absenteeism | Voluntarily; free of charge (paid by municipality: 50,000 euros) | 260 of 10,000 teachers | - | - | Although enthusiasm among Amsterdam teachers for the free flu shot was again not great this year, the municipality will offer it again next year. | |
| Het Parool, 2018[[11](#_ENREF_11)] | Netherlands | News article | Teachers of primary schools and high schools, and vocational schools | Reduce absenteeism; not possible to work from home; higher risk for teachers | Amsterdam: voluntarily; free of charge  Kapelle, Zeeland: voluntarily; bonus of 405 euros when vaccinated | - | Privacy; first look at own needs and then the collective; | - | “Using a free flu shot, Amsterdam tries to minimize absenteeism among teachers. Due to the teacher shortage, every teacher on sick leave, is one too much. But what if they do get sick? Is that still allowed?” | |
| Algemeen Dagblad, 2018 [[7](#_ENREF_7)] | Netherlands | News article | Primary school teachers | Reduce absenteeism |  |  |  |  | Virologist states: “From next year, all teachers should be vaccinated for the flu.” | |
| Gezondheid en wetenschap, 2017 [[4](#_ENREF_4)] | Belgium | News article | Primary school teachers | Reduce absenteeism | “Schools can decide whether they vaccinate their teachers, but it does not happen often as vaccines cost quite some money.” | - | - | Healthy adults 15-65y: number needed to vaccinate to prevent one case is 75 | “The effect of influenza vaccination in health people under the age of 65 is modest. The risk of getting the flu is small, and that small risk is reduced by half with a vaccination.” | |
| VOS/ABB, 2019 [[19](#_ENREF_19)] | Netherlands | News article | Employees with a lot of people-to-people contact; primary school teachers | Reduce absenteeism | Voluntarily; free of charge; different opinions about bonus | - | - | - | “Employers in education should reward their employees if they receive the flu shot in the fall.” | |
| Onderwijsland, [[1](#_ENREF_1)] | Netherlands | News article | School teachers | Reduce absenteeism; | Free of charge | - | - | “Scholen zouden goedkoper uit zijn door een griepprik aan te bieden, dan wanneer het op zoek moet naar vervangers voor de docenten.” | Virologist and schoolboard state: “Teachers should receive a free flu shot.” | |
| Nationale onderwijsgids, 2019[[13](#_ENREF_13)] | Netherlands | News article | Scholen voor persoonlijk onderwijs: teachers  Amsterdam: teachers of primary schools, high schools and vocational schools | Reduce absenteeism | Schools for personal education (Kapelle, Amsterdam, Geldermalsen, Hardegarijp, Deventer, Hengelo, Hoorn and Utrecht): bonus of 405 euro  Amsterdam: free of charge | Scholen voor persoonlijk onderwijs: 69% of 111 teachers  Amsterdam: 211 teachers (2.1%) | - | - | “A flu peak that lasted for months led to high absenteeism in education and healthcare. As a result, schoolboards and the municipality of Amsterdam have started experimenting with the flu shot.” | |
| Omroep Brabant, 2019 [[15](#_ENREF_15)] | Netherlands | News article | Primary school teachers | Reduce absenteeism | Amsterdam and Twente (mostly likely this refers to one school board in Twente, red.): free of charge |  | - | - | Virologist states: “Persons in front of the classroom are more susceptible to the flu. Therefore it would make sense for schools to vaccinate teachers.”  Schoolboard states: “It is worth it to consider a flu shot for teachers. There really must be a few hundred people sick for a long time if you really want to get in trouble. In addition, teachers are generally “very health people”. Exactly because they are in front of the classroom, teachers are often less susceptible to the flu. It is also their own responsibility.” | |
| Provinciale Zeeuwse Courant, 2018 [[12](#_ENREF_12)] | Netherlands | News article | High school teachers | Reduce absenteeism; relieve workload for colleagues | Schools for personal education (Kapelle, Amsterdam, Geldermalsen, Hardegarijp, Deventer, Hengelo, Hoorn and Utrecht): bonus of 405 euro | 31 of 37 teachers (84%) | - | - | “The coming months the schoolboard will keep a close eye on absenteeism due to illness. Polet: Only after the influenza peak we will know whether the bonus really resulted in fewer patients. Until then we have to wait.” | |
| Provinciale Zeeuwse Courant, 2018 [[10](#_ENREF_10)] | Netherlands | News article | High school teachers | Reduce absenteeism; relieve workload for colleagues | Schools for personal education (Kapelle, Amsterdam, Geldermalsen, Hardegarijp, Deventer, Hengelo, Hoorn and Utrecht): not free of charge, but bonus of 405 euro | - | - | - | “We believe that there is no point in taking measures that you know in advance will not be beneficial. The bonus is an experiment. We will evaluate the results and see whether we have fewer patients compared to the national average. | |
| Nationale Onderwjisgids, 2018 [[9](#_ENREF_9)] | Netherlands | News article | School teachers | Reduce absenteeism; relieve workload for colleagues | Schools for personal education ( Kapelle, Amsterdam, Geldermalsen, Hardegarijp, Deventer, Hengelo, Hoorn and Utrecht): not free of charge, but bonus of 405 euro | - | - | - | “At the end of this year, it will be examined whether at least a quarter of the teachers participated. In that case, the experiment will be continued. In two years’ time, it will then be examined whether absenteeism has been reduced more compared to the average in education. There are currently five Schools for Personal Education (Amsterdam, Geldermalsen, Hurdegaryp, Kapelle and Utrecht) participating. Next year, schools in Deventer, Hengelo and Hoorn will also start.” | |
| Christian Democratic Appeal (CDA), 2019 [[14](#_ENREF_14)] | Netherlands | News article | School teachers | Reduce absenteeism | Free of charge | - | - | - | “The Christian Democratic Appeal The Hague requests the city council to offer all teachers in The Hague a flu shot.” | |

***References***

1. Free flu vaccination for teachers [in Dutch: Gratis griepprik voor leraren]. Onderwijsland.

2. Seasonal influenza vaccination and antiviral use in Europe - Overview of vaccination recommendations and coverage rates in the EU Member States for the 2013-14 and 2014-15 influenza seasons. Stockholm: European Centre for Disease Prevention and Control, 2016.

3. Seasonal influenza vaccination in Europe. Vaccination recommendations and coverage rates in the EU Member States for eight influenza seasons: 2007-2008 to 2014-2015. Stockholm: European Centre for Disease Prevention and Control, 2017.

4. Should all teachers be vaccinated for the flu? [in Dutch: Moeten alle leerkrachten gevaccineerd worden tegen griep?]. Gezondheid en wetenschap. 2017.

5. Seasonal influenza vaccination and antiviral use in EU/EEA Member States. Stockholm: European Centre for Disease Prevention and Control, 2018.

6. Annual report public health service Amsterdam [in Dutch: Jaarverslag GGD Amsterdam]. GGD Amsterdam, 2018.

7. Virologist Ab Osterhaus calls for flu vaccination teachers [in Dutch: Viroloog Ab Osterhaus pleit voor griepprik docenten]. Algemeen Dagblad. 2018.

8. Municipality hands out free flu vaccination to teachers [in Dutch: Gemeente deelt gratis griepprik uit aan docenten]. Het Parool. 2018.

9. Schools for personal education experiments with reward for teachers who get flu vaccination [in Dutch: SvPO experimenteert met extra beloning voor docenten die griepprik halen]. Nationale Onderwijsgids. 2018.

10. Isaac Beeckman Academy in Kapelle gives teachers 405 euros bonus for flu vaccination [in Dutch: Isaac Beeckman Academie in Kapelle geeft docenten 405 euro bonus voor griepprik]. Provinciale Zeeuwse Courant. 2018.

11. Free flu vaccination for teachers: are they allowed to be ill? [in Dutch: Gratis griepprik voor leraren: mogen zij wel ziek zijn?]. Het Parool. 2018.

12. Bonus for flu vaccination tempts teachers at Isaac Beeckman Academy [in Dutch: Bonus voor griepprik verleidt docenten Isaac Beeckman Academie]. Provinciale Zeeuwse Courant. 2018.

13. Flu epidemic 2019: whether or not a flu vaccination for teachers? [in Dutch: Griepepidemie 2019: wel of geen griepprik voor leraren?]. Nationale Onderwijsgids. 2019.

14. Christian Democratic Appeal The Hague wants a flu vaccination for all teachers [in Dutch: CDA Den Haag wil voor alle leraren een griepprik]2019.

15. Brabant O. Just give teachers a flu vaccination, says this virologist [in Dutch: Geef juf en mees gewoon een griepprik, zegt deze viroloog]. Omroep Brabant. 2019.

16. Free flu vaccination for teachers Amsterdam [in Dutch: Gratis griepprik voor Amsterdamse leraren]. Het Parool. 2019.

17. Flu vaccination for teachers Amsterdam also in 2020 [in Dutch: Griepprik voor Amsterdamse leraren ook in 2020]. Het Parool. 2019.

18. The annual influenza vaccination [in Dutch: De jaarlijkse griepvaccinatie]: RIVM; [updated 04-02-202017-04-2020]. Available from: <https://www.rivm.nl/griep-griepprik/zorg/vaccinaties/elkjaar>.

19. Reward employees who get a flu vaccination [in Dutch: Beloon werknemers die griepprik halen]. VOSABB. 2018.

20. Gezondheidsraad. Offer advice "Flu vaccination: revision of assessment" [in Dutch: Aanbieding advies "Griepvaccinatie: herziening van de indicatiestelling"]. 2007.

21. Gezondheidsraad. Advisory letter Vaccination seasonal flu [in Dutch: Briefadvies Vaccinatie tegen seizoensgriep]. 2011.

22. Staatssecretaris van Volksgezondheid Welzijn en Sport. Request for advice influenza [in Dutch: Adviesaanvraag influenza]. 2018.

23. Gezondheidsraad. Work program 2020 Health Council of the Netherlands [in Dutch: Werkprogramma 2020 Gezondheidsraad]. Den Haag: 2019.
