# Supplementary material for: Influenza vaccination of school teachers: A scoping review and an impact estimation
Source: PLoS One. 2022 Aug 11;17(8):e0272332. doi: 10.1371/journal.pone.0272332 (PMC9371289; doi:10.1371/journal.pone.0272332)
Supplement: S3 File — (DOCX) [file pone.0272332.s003.docx]

**S3 Supplementary. Interviews**

This supplement provides the following sections:

***Methods, Informed consent, Interview guide, Results, and 1 Table.***

***Methods***

Interviews were conducted to obtain in-depth information from key informants from two Dutch municipalities encompassing the two largest cities (Amsterdam and Rotterdam). The questionnaire was semi-structured, containing open-ended questions to allow for discussion, supplemented with closed questions if the reply did not fully cover the topic. Interviewees were selected based on their professional experience with influenza vaccination and/or their possible involvement in or consideration of vaccination of school teachers. They were identified through contacts with the PHS in Amsterdam and Rotterdam. Potential participants were approached by email and they received a letter asking whether they would agree to participate. The letter explained research aims, interview procedures and privacy matters (de-identification of transcripts). Written and oral consent was obtained from each consenting interviewee (see below for translated informed consent form). The consent form included queries on whether the interviewee had read the information letter and whether all their queries and concerns had been addressed satisfactorily. Ethical approval was not required for this study according to Dutch legislation, as it concerned a once only interview. Consenting participants were interviewed via telephone conversation between 22 April 2020 and 16 June 2020 as face-to-face interviews were not possible due to COVID-19 epidemic lockdown. All interviews were recorded and then transcribed and analysed by one researcher (AH) using ATLAS.ti version 8.3.20.0 and an inductive coding strategy (using themes that emerged from raw data) ([1](#_ENREF_1)).

***Informed consent***

*Subject: participation in the study “Influenza-vaccination of teachers: what are the considerations and what is the possible impact?*

Hereby I declare to have read the information letter. I fully understand the content of this document and I have been able to ask all my questions. I am satisfied with how they were answered. Also, I have had sufficient time to consider my decision to participate in this study.

I understand that my participation in this study is voluntarily and that I can end my participation at any given time, without having to motivate this choice.

I am fully aware of the fact that involved researchers know my name and function but will not use this.

I hereby give permission to use my anonymized information for the purposes described in the information letter. This includes a possible publication in a scientific journal.

Name:

Signature: Date: __ / __ / __

***Interview guide***

General

Date of interview:

Current position:

Age:

Sex:

Introduction

My name is Anne Huiberts and I am a master student at the Radboud University Nijmegen. I study Biomedical Sciences. Currently, I’m doing my internship at the Dutch National Institute for Public Health and the Environment and studying influenza vaccination for school teachers. I focus mainly on the considerations to vaccinate teachers and estimating the possible impact. In order to do so, I conduct interviews with experts in the field, conduct a literature review of both grey and scientific literature, and using newspaper monitoring I will make inventory of which regions in the Netherlands offer influenza vaccination for teachers, or have considered this. I would like to thank you taking the time to share your experiences and opinions with me.

Start question

Is an influenza vaccination offered to teachers in your municipality? (apart from the medical risk groups who are approached by the general practitioner to get their vaccination)

Questions in case influenza vaccination for teachers is offered:

**Origin of plan / policy**

1. Can you tell me about the first time you heard about the idea to start offering influenza vaccination to teachers?
   1. From who did you hear this?
   2. Where did you hear about the idea?
   3. How did you hear about the idea?
2. Do you know how it got from idea to policy? Can you tell about this?
   1. Can you describe the process?
   2. Who were involved?
      1. Were schoolboards and/or teachers involved?
   3. When did the idea come up and when did the preparations start?
   4. Is the advice of the municipal health service included in the decision? Were there conflicting interests between municipality and municipal health service? Who makes the final decision on policy?

**Reason for implementing policy and its purpose**

1. Can you explain to me why [in your municipality] influenza vaccination is offered to teachers?
2. Can you tell me what you think the main goal of the municipality was to vaccinate teachers for influenza?
3. What do you think is the main goal of vaccinating teachers?

**Implementation of policy**

1. Can you explain how influenza vaccination is organized within this target group?
   1. Who/which body offers the vaccination?
   2. To which teachers/school types is the vaccination offered?
   3. How are schoolboards and teachers informed about the vaccination?
   4. How are teachers invited to the vaccination?
   5. Where are the vaccinations given?
   6. Who gives the vaccinations?
   7. How long does the campaign take?
   8. How many nurses or other qualified people are needed to give all these vaccinations?
   9. Do you know what the costs are for vaccinating this target group and who is funding it?
2. Is this vaccination free for teachers? (yes/no/partly: for whom and for whom not)
3. Is it a voluntary or mandatory vaccination?
   1. Was mandatory vaccination considered? Why?
   2. Do teachers receive a compensation when they get vaccination? Has it been considered?
4. Do you know what percentage of teachers takes the flu vaccination?
   1. What do you think of this?
   2. What do you think are the main reasons for teachers to get vaccinated?
   3. What do you think are the main reasons for teachers not to get vaccinated?
   4. Do teachers who have not attended receive a reminder?
   5. Do you have any ideas on how vaccination uptake could be increased?
   6. Do you expect teachers’ willingness to change now that Sars-CoV-2 can also coincide with influenza infections?

**Evaluation of the policy**

1. What are the benefits of influenza vaccination for this target group?
   1. What do you think are the benefits of vaccinating teachers?
   2. What do you think [other] municipal health service employees consider benefits of vaccinating school teachers?
2. What are the disadvantages of influenza vaccination for this target group?
   1. What do you think are the disadvantages of vaccinating teachers?
   2. What do you think [other] municipal health service employees consider disadvantages of vaccinating school teachers?
3. Can you describe the acceptance of influenza vaccination by this target group?
   1. Do you agree with the policy of your municipality?
   2. Do the pros or cons of influenza vaccination outweigh for this target group?
   3. What do you think [other] municipal health service employees think of vaccination of this target group?
4. How do you think teachers feel about the influenza vaccination?
5. How much absenteeism among teachers do you think the influenza vaccination can prevent?
   1. If all teachers are vaccinated?
   2. With the current uptake?

**Other municipalities/regions**

1. Do you know of other regions
   1. that offer influenza vaccination to teachers?
   2. that deliberately do not offer influenza vaccination to teachers after consideration?

**Closing**

1. Are there any other topics that you consider relevant to the research that have not been addressed during this interview and that you would like to say anything about?
   1. If you remember something after the interview, you can always contact me.
2. Do you have any questions for me following the interview?

Questions in case influenza vaccination for teachers is not offered:

**Considerations**

1. Did you ever discuss vaccinating teachers for influenza with your colleagues?
2. Has vaccinating teachers for the flu been considered by your municipality or municipal health service?
   1. Can you describe the process?
   2. Who were involved?
      1. Were schoolboards and/or teachers involved?
   3. When did this take place? As a result of which?
   4. Is the advice of the municipal health service included in the decision? Who makes the final decision on policy?

**Reason for not implementing policy**

1. What are the reasons for your municipality not to vaccinate this target group?
2. Do you think that teachers in you municipality should be vaccinated?
   1. What do you think are the benefits of vaccinating teachers?
   2. What do you think are the disadvantages of vaccinating teachers?
3. [If yes] How would envisage the organization of influenza vaccination for teachers?
   1. Should vaccination be free or should teachers pay for it themselves? (yes / no / partly: for whom and who not)
   2. Should vaccination be voluntary or mandatory?
   3. Do you that the teachers who get the vaccination should get a compensation?
   4. Which teachers / school types should it be offered to?
   5. Should teachers arrange this themselves or should it be organized by another party?
      1. By whom / which body?
      2. How? (call schoolboards and teachers, giving the vaccinations)
      3. Who should fund it [if free vaccination]?

**Evaluation**

1. What do you think [other] municipal health service employees of not offering influenza vaccination for this target group?
2. How do you think teachers feel about the influenza vaccination?
   1. Do you expect teachers’ willingness to change now that Sars-CoV-2 can also coincide with influenza infections?
3. What percentage teachers would take the free vaccine do you think?
4. How much absenteeism among teachers do you think the influenza vaccination can prevent [if all teachers take the vaccination]?

**Other municipalities/regions**

1. Do you know of other regions that offer influenza vaccination to teachers?
   1. What do you think of this?
   2. Do you know of other regions that deliberately do not offer influenza vaccination to teachers after consideration?

**Closing**

1. Are there any other topics that you consider relevant to the research that have not been addressed during this interview and that you would like to say anything about?
   1. If you remember something after the interview, you can always contact me.
2. Do you have any questions for me following the interview?

Questions for school principals in case influenza vaccination for teachers is offered:

**Origin of plan / policy**

1. Can you tell me about the first time you heard about the idea to start offering influenza vaccination to teachers?
   1. From who did you hear this?
   2. Where did you hear about the idea?
   3. How did you hear about the idea?
   4. Were schoolboards and/or school principals and/or teachers involved in the implementation of the policy? Or were they just informed after the policy was implemented?

**Reason for implementing policy and its purpose**

1. Can you explain to me why [in your municipality] influenza vaccination is offered to teachers?
2. Can you tell me what you think the main goal of the municipality was to vaccinate teachers for influenza?
3. What do you, as school principal, think is the main goal of vaccinating teachers?

**Implementation of policy**

1. Can you explain how influenza vaccination is organized within this target group?
   1. How are schoolboards, school principals, and teachers informed about the vaccination?
   2. How are teachers invited to the vaccination? (by the municipal health service, the role of schoolboards/principals)
   3. Who gives the vaccinations?
   4. Where are the vaccinations given?
   5. What would you, as school principal, do if influenza vaccination is not offered anymore by the municipal health service? (stop offering it, or arrange it in a different way, or just advice)
2. Is this vaccination free for teachers? (yes/no/partly: for whom and for whom not)
3. Is it a voluntary or mandatory vaccination?
   1. Do teachers receive a compensation when they get vaccination? Has it been considered?
4. Do you know what percentage of teachers at your school takes the flu vaccination?
   1. What do you think of this?
   2. What do you think are the main reasons for teachers to get vaccinated?
   3. What do you think are the main reasons for teachers not to get vaccinated?
   4. Do teachers who have not attended receive a reminder?
   5. Do you have any ideas on how vaccination uptake could be increased?
   6. Do you expect teachers’ willingness to change now that Sars-CoV-2 can also coincide with influenza infections?

**Evaluation of the policy**

1. What are the benefits of influenza vaccination for this target group?
   1. What do you think are the benefits of vaccinating teachers?
   2. What do you think schools consider benefits of vaccinating school teachers?
2. What are the disadvantages of influenza vaccination for this target group?
   1. What do you think are the disadvantages of vaccinating teachers?
   2. What do you think schools consider disadvantages of vaccinating school teachers?
3. Can you describe the acceptance of influenza vaccination by this target group?
   1. Do you agree with the policy of your municipality?
   2. Do the pros or cons of influenza vaccination outweigh for this target group?
   3. What do you think other schools think of vaccination of this target group? Did you discuss this with other schools?
4. How do you think teachers feel about the influenza vaccination?
5. How much absenteeism among teachers do you think the influenza vaccination can prevent?
   1. If all teachers are vaccinated?
   2. With the current uptake?

**Other municipalities/regions**

1. Do you know of other regions
   1. that offer influenza vaccination to teachers?
   2. that deliberately do not offer influenza vaccination to teachers after consideration?

**Closing**

1. Are there any other topics that you consider relevant to the research that have not been addressed during this interview and that you would like to say anything about?
   1. If you remember something after the interview, you can always contact me.
2. Do you have any questions for me following the interview?

Questions for school principals in case influenza vaccination for teachers is not offered:

**Considerations**

1. Did you ever discuss vaccinating teachers for influenza with your colleagues?
2. Has vaccinating teachers for the flu been considered by your municipality or municipal health service?

**Reason for not implementing policy**

1. What are the reasons for your municipality not to vaccinate this target group?
2. Do you think that teachers in you municipality should be vaccinated?
   1. What do you think are the benefits of vaccinating teachers?
   2. What do you think are the disadvantages of vaccinating teachers?
   3. What are reasons for you not to offer your personnel an influenza vaccination?
3. [If yes] How would envisage the organization of influenza vaccination for teachers?
   1. Should vaccination be free or should teachers pay for it themselves? (yes / no / partly: for whom and who not)
   2. Should vaccination be voluntary or mandatory?
   3. Do you that the teachers who get the vaccination should get a compensation?
   4. Should teachers arrange this themselves or should it be organized by another party?
      1. By whom / which body?
      2. How? (call schoolboards and teachers, giving the vaccinations)
   5. Which teachers / school types should it be offered to?
   6. Who should fund it [if free vaccination [if free vaccination]?

**Evaluation**

1. What do you think other schools/school principals think of offering influenza vaccination for this target group? Did you discuss this with other schools?
2. How do you think teachers feel about the influenza vaccination?
   1. Do you expect teachers’ willingness to change now that Sars-CoV-2 can also coincide with influenza infections?
3. What percentage teachers would take the free vaccine do you think?
4. How much absenteeism among teachers do you think the influenza vaccination can prevent [if all teachers take the vaccination]?

**Other municipalities/regions**

1. Do you know of other regions
   1. that offer influenza vaccination to teachers?
   2. that deliberately do not offer influenza vaccination to teachers after consideration?

**Closing**

1. Are there any other topics that you consider relevant to the research that have not been addressed during this interview and that you would like to say anything about?
   1. If you remember something after the interview, you can always contact me.
2. Do you have any questions for me following the interview?

***Results***

*Characteristics of interviewees.* Ten participants (3 men and 7 women) in Amsterdam (n=5) and Rotterdam (n=5) with an average age of 48 (range 37 – 61) were interviewed via a phone call. These were PHS physicians (n=3), policy officers (n=4), school principals (n=2) and a manager in youth healthcare (n=1). Excerpts of the interviews are shown below in table S3.1.

*Implementation of influenza vaccination for teachers.* Implementation of teacher influenza vaccination in Amsterdam was mostly described as a political decision to minimize teacher shortage with a lack of evidence-base that had been hastily set up. It was also considered a matter for occupational health rather than public health. Rotterdam decided not to offer free vaccination to teachers after consultation with school boards. Interviewees gave different reasons why it was not implemented (possible limited effect, costs, potential teacher resistance, privacy issues, lack of time, too large organisational effort and possible low uptake). After our invitation, but prior to actual interview, one interviewee had requested information from eight large schoolboards in Rotterdam on whether influenza vaccination was offered to their personnel. Of those eight schoolboards, some had not discussed this topic (n=3), some did not offer it after consideration (n=2) and some did offer or had offered it (n=3). The two schoolboards that consciously did not offer influenza vaccination to teachers mentioned limited effect, privacy issues, too large organisational effort and possible low uptake as main arguments. The three schoolboards that currently or previously offer vaccination also described a low uptake (without mention of specific uptake rates).

*Organisation.* In Amsterdam, the teachers vaccination campaign was financed by the municipality and executed by the PHS at the PHS (2018/2019 and 2019/2020). Vaccination was intended for all school personnel (primary schools, secondary schools and vocational high schools) and concerned a free and voluntary influenza vaccination for which they were invited on one of the two designated evenings at the municipal health centre, since attending all schools separately would be too labour-intensive and be privacy sensitive (i.e. it would be visible for school staff which teachers do and do not take the vaccination). Schoolboards were expected to forward the information about the vaccination campaign to individual school managers and teachers. A bonus for those who received their vaccination was not considered and some interviewees (n=3) also describe this as undesirable or not necessary. The majority of respondents from Rotterdam propose offering flu vaccination with a broad approach, including both regular education and education of children with special needs, primary and secondary schools (n=4). Two respondents mentioned including pre-school care, and after school-hours day care. One respondent thought schools for children with special needs already offered vaccination to teachers, as they work with a vulnerable group. Interviewees proposed that occupational physicians (n=5), general practitioners (n=3) and private institutes (n=1) could provide influenza vaccination for teachers, in addition to the municipal health service (n=3). Accessibility of the municipal health service due to its location in the centre of Amsterdam was considered problematic, negatively affecting vaccination uptake (n=4). Additionally, respondents proposed better communication with teachers, as many may not have heard of the vaccination program. Three interviewees described that offering an influenza vaccination to teachers is the responsibility, including financial responsibility, for schoolboards together with their occupation physicians, not that of the municipality.

*Teachers’ attitude.* No systematic evaluation was performed among the teachers offered vaccination in Amsterdam, but most frequently mentioned by interviewees as the expected motivation for uptake was protecting one’s own health (n=6) and for refusing uptake being that the government should not involve itself in a personal health issue (n=5). Other possible reasoning for receiving the vaccination was protecting the health of others (n=3) and ‘no harm in trying’ (n=2). Other reasons reported by key informants for refusing uptake included teachers being young and healthy (n=3), logistical considerations (n=3), fear that the vaccination can make you ill (n=2), needle anxiety (n=2), not knowing about it / no interest (n=2) and religious considerations (n=1).

*Advantages and disadvantages of vaccinating school teachers.* Different advantages of influenza vaccination for teachers were described: protection of students/others (n=2), protection of one-self (n=2), reducing teacher absenteeism (n=2), herd immunity (n=2) and teacher mental well-being (n=1). More detail in S3 supplementary. Advantages were expected to differ per school type; for some schools reducing teacher absenteeism was expected to be the largest advantage and for other schools (for children with special needs) this was expected to be protecting vulnerable students by reducing the risk of transmission (n=1). Also per school-level advantages were expected to differ: at the individual level one’s own health and that of others might be most important, while at school-level the most important advantage can be reducing teacher absenteeism and on societal level it could be reaching herd immunity (n=1). Apart from (the misconception of) being vulnerable for other infectious diseases in the week after vaccination (n=1) and some people not being able to receive a vaccination due to contra-indications (n=1), no disadvantages of vaccinating teachers for influenza were mentioned by the interviewees.

*Vaccination uptake.* Overall vaccination uptake in Amsterdam was expected to be low (10% was expected in 2018/2019 and 4% in 2019/2020). The first year this expectation was based on the 10% vaccination uptake of the influenza vaccination offered by the municipal health centre to its own personnel. The following year this was adjusted downwards based on the actual vaccination uptake by teachers in the first year. At only 2%, this actual teacher VU in Amsterdam was lower than expected. A hasty set-up and poor communication (probably not reaching teachers) were given as reasons. For one school in Amsterdam at least two teachers had received the vaccination at the municipal health centre (6% of staff) and 29% of staff was expected to have received it through their risk group status. Turn-up by risk group for the entire program was unknown; those attending could have belonged to a risk group. The expectations in Rotterdam towards a hypothetical program varied: two respondents expected a low VU, as teachers were perceived as young and healthy, and one respondent expected at least a 50% uptake. Schoolboards offering vaccination in Rotterdam described a low uptake (no specific rates mentioned except for one board: <1% uptake). Suggestions for increasing the VU in Amsterdam and Rotterdam were health education (n=3) and better practical organization with easy accessibility (n=2). An interviewee from Amsterdam described the low influenza vaccination uptake in the teacher population as “ethically not important” as there was no medical necessity.

*Evaluation.* Effectiveness of influenza vaccination for teachers was expected to be low (n=5). The other five respondents did not know or had too little information to make an estimation of the effectiveness. Of this group, one did mention that effectiveness of influenza vaccination is high but that it would be hard to measure the effect on absenteeism. The municipal health centre staff experienced it as very labor-intensive, while the municipality expected higher labor-intensiveness if the program was to be performed through occupational health services.

Multiple interviewees (n=4) describe the privacy sensitive nature of offering influenza vaccination to teachers, or vaccination in general. The municipality in Amsterdam, for this reason among others, decided not to offer it on-site at schools and not to register who received the vaccination. The privacy issues were expected through the visibility for other staff of those taking or refusing the vaccine. Subsequent sick-leave might then be viewed negatively by staff or employers. Also one respondent and one of the Rotterdam schoolboards therefore decided not to put pressure on taking the vaccination. They described it as teachers own responsibility and the necessity to let them make their own choice.

Two interviewees were of the opinion that reducing absenteeism should never be the reason to implement influenza vaccination. They described vaccination as something you receive because of a health risk, not because of benefits for your employer. Some interviewees expressed vaccination not being the right approach to the teacher shortage.

*Effect of COVID-19.* The effect of the COVID-19 epidemic on the willingness to receive influenza vaccination and the attention for influenza vaccination was described in different ways by interviewees. The majority (n=6) expected an increase in teacher influenza vaccination uptake in the next season due to the COVID-19 pandemic. Motivations were the ‘realizing one’s vulnerability’ and the ability to at least protect oneself against the flu; the importance of vaccination as a virus can shut down a society; and to make it easier to know when one is infected by Sars-CoV-2. One interviewee referred to the 2009 pandemic influenza A (H1N1)pdm09 outbreak which in the year after resulted in more people receiving the seasonal influenza vaccination. Three other interviewees pointed out that Sars-CoV-2 is very different from flu and they expected limited effect on vaccination uptake. Of those three, one interviewee even foresees less attention, interest and time for influenza vaccination due to the COVID-19 pandemic and a possible Sars-CoV-2 vaccine. Next to the effect on willingness and attention for influenza vaccination as a result of the COVID-19 pandemic, one interviewee expects it to translate in less people working while ill, thereby also reducing transmission of influenza.

*Scientific evidence and national policy advice*. Half of the interviewees (n=5) indicated that scientific evidence on the utility, necessity and effectiveness of vaccinating teachers against influenza and therefore sound argumentation to implement it, is currently lacking. Two interviewees explicitly mentioned their doubt whether teachers have a higher risk for influenza. Three interviewees mentioned that influenza vaccination for teachers is currently not advised by the Health Council. When there would be an advice describing teachers as a risk group for influenza or describing a societal or national interest of vaccinating teachers for influenza, organising influenza vaccination for teachers would be a public health issue and more appropriately organized by the municipality instead of other parties (n=2).

*Knowledge about other regions.* The majority of the interviewees did not have knowledge of other regions in the Netherlands that offer influenza vaccination for teachers (n=7).

*Local initiatives*. Our interviews found that Rotterdam school boards considered free influenza vaccination for their teachers, and some even implemented it. These school boards did not appear in our newspaper search, and therefore, it is to be expected that more school boards in the country offer their personnel influenza vaccination. Two foreign news articles (Australia and UK) also illustrate that, although teacher influenza vaccination is neither advised nor offered for free at a national scale, it is sometimes organized locally ([2-5](#_ENREF_2)).

***Table***

Table S3.1. Excerpts from the interviews (English translation following each Dutch quote).

| **Subject** | **Excerpt from interview (translated Dutch to English)** |
| --- | --- |
| Hearing about idea/ implementation of flu shot for teachers | A somewhat remarkable route. (interview 1) |
|  | That actually came in through a very remarkable, or a bit strange way. (interview 2) |
|  | That I sometimes think it is quite strange that a politician who is not medically trained, can decide whether or not to make it available .. or actually .. give permission for the vaccine, because the vaccine is still a medicine. Uhm yes you know, it is preventative and for prevention everyone always has to do everything .. well … it is accepted quickly. It is possible, say we just make antibiotics available to all teachers, because maybe they will have diarrhea and cannot come to school, they can take antibiotics. That will be a completely different discussion, and then I think, yes, of course that is different, but it is still a medicine we are talking about. That is .. at least, it fascinates me. (interview 4) |
|  | What I at least found a pity at the municipality of Amsterdam is that there is certainly not .. that it was done purely for political reasons, but no further thought was given to what would be achieved. Certainly with such low turnouts you have of course .. well the profit is really minimal while it takes a lot of time and energy. And money. I am disappointed that it was not considered better. And I find it somewhat opposed to the advice of the national Health Council. Because in principle we follow the advice of the Health Council as how they are implemented and they at least do not describe teachers as a risk group. So yes .. it should have been more .. not just a political but also a medically related decision, I think. But that’s my opinion. (interview 4) |
|  | So it .. it is not based on ignorance, but actually in most cases .. yes most have indeed .. almost all have considered it, of which have offered it. Only the support of teachers themselves seems to be very low. (interview 10) |
| Reason for (no) implementation | Purely as extra gesture to the teachers, in the context of the enormous shortages. And we always know that there will be a flu peak, so many schools are already under pressure. Classes are being sent home. No it is also different than it was, but all right. It was already almost impossible to manage for many schools and teachers. We had foreseen, that if the influenza peak would hit, schools would literally fall apart. And then we thought, well, “no harm in trying, we just offer this” and yes, really more from that point of view, really from the teacher shortage. (interview 3) |
|  | Well, I try to look at it from a teachers or employees point of view. Suppose my employer would say, well [name] you get the flu shot and you also get 405 euros. Then I think “gats”, bit of bribery. But you do that because of a risk you have, or because of your health, but not because my employer wants it so badly. (interview 2) |
|  | The most important purpose, is to protect another. Well, if you find that very difficult, then to protect yourself. And then it is a bonus if you don’t happen to get sick when you do your work. But if that is the goal, not to get sick at work ..! Yes, then I think “you are not indispensable”, because if people are indispensable, then you really have to do something about it. (interview 9) |
|  | Uhm and it is also done as kind of support, making up for the teacher shortage. I can imagine, but not sure, that teachers may have also had the feeling that it’s better to buy something with the money, buy teachers for it or train teachers for it instead of offering a vaccine. (interview 4) |
| Effect of covid19 | I think that it will certainly play a role, yes! I can imagine that very well. That people think .. also parents who may not vaccinate their children now for all kinds of reasons, that they will do it now. But maybe not, you don’t know. There are also stories that people find [unintelligible] and danger and all.  For example, I could imagine now that in due to corona, that it is said “yes .. later”, I do not know if it’s true, but I can imagine something like that, that they say “it might be handy if you’re at least vaccinated for the flu, that if you get symptoms, well then you have .. then you know it is not the flu. (interview 2) |
|  | Given what’s going on right now .. I think the municipality health centre has something else on its mind, as do we. What we are looking for is a different vaccine rather than the annual flu vaccine.  Look, we know that the flu vaccine doesn’t protect against corona, so I imagine you’re thinking “what do I get that shot for”. (interview 3) |
|  | Yes, maybe people realize that every person is still vulnerable and can get sick and they think, well let’s just do it, because then at least it will not be that. (interview 4) |
|  | Yes, I hope so! Because you hear everyone about a vaccine for corona, and then I think “that’s great, go and get those other vaccines for your children too then! So yes, I think there is more awareness that such a vaccine is useful. (interview 6) |
|  | Yes, that is a nice actuality of course. If you can indeed demonstrate with research that vaccination for these types of influenza forms, that it really has an effect. Yes, then I think that, especially with what has now happened in mind, many people are quite open to doing that. (interview 7) |
|  | Well, I think .. if they are well informed, I think it should not make a difference. […] Well, because corona is of course very different from the flu virus. (interview 8) |
|  | Well, I think .. you can, you often see it after a major outbreak, with H1N1 it was also true that many more people came to get a vaccination in the following year. You could explain that: at least we can think of corona when she gets a fever rather than think of influenza […]. But yes, you can never rule it out, so you should always investigate both.  I know, let’s put it this way, hopefully, at least that is what I think, after the corona period people will not dare come to work with a fever. Very pleasant. Because that always infects someone. That just .. you see it very often. One is coughing and five days later the next one is coughing. Then the next and before you know it the whole department has gone through it once. That is simply unpleasant. (interview 9) |
|  | Actually, you only see “I don’t know what will be the effect”, not that they express a kind of expectation, because of course I have also asked whether they would consider it. (interview 10) |
| Communication about flu shot | But we also received a lot of phone calls afterwards, from teachers saying “yes, but I haven’t had it at all”. Because we communicated this via the schoolboards and asked them to forward it to their schools, and then [unintelligible] it also happened that a school principal thought it was nonsense and did not forward it at all. Yes ..  But it is very contradictory that there are a lot of teachers .. or a lot, multiple teachers who called us afterwards like “yes, hoe zit dat nou”. Because they had read it in the newspaper, but received no oproep. Yes, then you had to say “yes I don’t know, it should have reached your principal, so I don’t know”. (interview 1) |
|  | Afterwards we heard that many teachers have not received that letter at all. That it did not come through, with a schoolboard that is supposed to forward it to the school management, which then has to forward it to the teachers. So I think, if I ever start with something, and we want to reach teachers. Then we will approach the schools directly, and not through a schoolboard. (interview 2) |
| No attention / interest in education for flu shot | Yes, it’s a shame that if it’s offered to you … it is already offered when you’re older than 60, so you don’t even have to be unhealthy, but people .. “no, because I am never sick”, “yes ..” “no, I’m never ill so I don’t have to” “but you’re over 60” “no but I never get ill”. Yes well, then I thing, should you then explain again what the disadvantages are if they do get ill … they are not impressed, they never hear about it. For example, about corona people already say “you know anyone who has had corona, I don’t know anyone”. A lot of people that say and think “what are we talking about”. And that’s already true for corona, let alone … while that has even been on television, that people can get in need of intubation. That’s possible with influenza too! But that .. you never get to see that on television so … people don’t think about that at all. (interview 9) |
|  | No, there is actually no attention for it in the educational field around me. (interview 7) |
| Target group / teachers as risk group | Yes, and it’s not only the teachers. We’ve said school staff, so also the concierge and .. they can come too. (interview 3) |
|  | I wonder .. maybe because they work with younger children who touch each other more often. It also depends on how the ventilation in the classes is of course. It’s all mechanically controlled nowadays, well I’m not an expert. Well yes, I’m not sure whether it is higher. (interview 9) |
|  | Yes, if it has to do with children, if that’s the underlying idea, then I think you would come across all forms of education where you have intensive contact with children. But then I am also thinking of childcare, for example, and before school. So toddlers, little toddlers, babies. So those target groups I think of in first instance, and if it turns out to be relevant to add secondary school or vocational education, you should definitely do that too. Only yes of course, how relevant it is I cannot estimate properly. I find that difficult. (interview 7) |
|  | Well I would offer it for all school types. Because also for regular education it would be nice if teachers don’t get ill. In any case it is nice if they do not get ill, as the educational sector has often troubles with shortages. It would be good if it could be offered broadly, but I think the advantages will differ per branch. [unintelligible] For one it is the absenteeism and for the other the much smaller risk of infection. (interview 6) |
|  | Hm .. no because look, small children who .. are known to take the influenza virus from home to school, because those children play with each other, which .. yes, so you start with the lower classes … the lowest classes actually, the classes, nurseries. And then yes, eventually you will end with, if it turns out to be popular, you end with the secondary schools and those will have little less need I think, but then .. then you can offer it. (interview 9) |
| Vaccination coverage | And we have, I see here, that we expected that … we called 9000 teachers and that a maximum of 1000 people would show up. That turned out to be much less and in the end 211 teachers came. And 9970 were called up, so we had a turnout of 2.1%. Joehoe! And we organized three evenings in 2018. (interview 1) |
|  | And the first year we had … where do I have those numbers of last year .. there we estimated what percentage will we vaccinate .. because we also give the employees of the municipal health centre the opportunity to get vaccinated, so we act as (and with “we” I mean the department of infectious diseases) as a sort of occupational health service. Like if you want to get vaccinated or you actually should attend the general practitioner but you don’t have time, you can come to us. And we looked what percentage is vaccinated at the municipal health centre. Well that we have .. we have applied that calculation to the teachers and ordered so many vaccines. (interview 2) |
|  | But yes, if you look at the proportion, then it really is only a small percentage that shows up. And that can .. I don’t know who came of course .. that can also be the older teachers. No idea what’s behind it all. (interview 3) |
|  | But medically there was no necessity, so I don’t think it’s interesting at all from an ethical point of view. (interview 1) |
|  | it also took a lot of effort (interview 5) |
|  | Well it could be .. so those people who visit their general practitioner will not come anyway. I think a maximum of 7 percent. (interview 9) |
|  | I know it differs a lot per school type, because those schools that I just mentioned where really sick children go, I think the willingness would be much higher there. But I think that at a regular primary school you will at least reach half, but whether that is 70 or 90 percent… (interview 6) |
| Privacy | In addition, when we offer it as an employer and people choose not to make use of it, then in this group, or within the team, it could lead to skewed reactions if they subsequently drop out because of the flu. (interview 10) |
|  | We made a conscious choice then, we are definitely not going to organize it at those schools, as this could lead to a bit of a strange atmosphere with those teachers and you want to prevent that of course. (interview 3) |
|  | No, but you know what I do think that vaccination, yes it’s always .. the choice is always up to the persons themselves to do that and yes you know I … I don’t want to fight about “you should” or “you will”, because of course we will get that a much worse situation with corona. Then we get corona vaccination and then people refuse that and then what, you know, and then someone gets sick and then what? You know, you had the chance to get vaccinated, you didn’t do it and you get sick. I don’t want to be pushed into that position that I have to have an opinion about that, because that’s not my job. |
|  | I can imagine that they consider it to be a personal consideration. So that you, yes that you do not want to interfere in that as a municipality, in that personal domain. (interview 8) |
| Executive party | We would not just organize this ourselves as municipality, because we really look at where the primary responsibility lies. If you “organize it away” from an organization that properly has to deal with “how high is my absenteeism, what is the cause, is it the workload or is it indeed other things”, we would not simply take it from them. So we would always pick the route where schoolboards have to do that and then based on this evaluation we would not organize it. (interview 10). |
|  | I like, what I like is that the occupational health service will start doing it, because it really is an occupational health task. I really think that, as it is about taking care of your own staff, so that is an occupational health issue. And in that sense, I think schools can better make their own consideration, which they then discuss with their own occupation health service like “I do want to offer a flu shot to the teachers” or not. I am curious whether the education department will let the general vaccination, which of course they had as we called everyone on, whether they will now give it back to the schoolboards. At least they will get more freedom to organize it themselves. Yes, it is debatable how that will work out, and whether the schoolboards not just say “well I don’t feel like organizing this with my occupational health service so..”. (interview 1) |
|  | Let me think .. yes, if I could think of anyone else than it would be the occupational physician. Uhm .. that could do that .. or yes .. or just at the general practitioner. (interview 6) |
|  | And then it should be offered by the employer, and .. but than you get reactions like “yes that is because I am not allowed to get sick from work, isn’t it” so that would be annoying too I would say .. then so to say, apart from the fact that we do not have enough people to do it, they could come to the municipal health centre to get vaccinated, voluntarily. But the point is, if you have to let people do something, you have to go there, they will not come to you. So as municipal health centre you have to vaccinate on location. You could offer that. (interview 9) |
|  | No, I think you should be able to do that at your own general practitioner. Every general practitioner has its own timeslots for flu shots and I think that if teachers would be able to make use of those, that they are simply able to register for it, and hand in a register form at the municipal health service or something, I don’t know, because of the costs. (interview 5) |
| Bonus when receiving vaccination | Then I think “gats”, bit of bribery. But you do that because of a risk you have, or because of your health, but not because my employer wants it so badly. (interview 2) |
|  | No! No. I find that very undesirable. (interview 6) |
| Obligation / voluntarily | Well I don’t know how that could be. We at least did not consider it, since we did not see the medically necessity at all. And from the city .. look, schoolboards are not under control of the stadsbestuur, so the city cannot decide on this. Then it would have to be on national level. Well I don’t really see that decision coming anytime soon. (interview 1) |
|  | I have not heard of it, and it does not seem true to me. There is not one vaccination obligated in the Netherlands, and I don’t even think it is possible according to the law. (interview 2) |
| Free vaccination | Uh, I think that if they have to pay for it themselves, it will put a lot of pressure on the willingness to do it. Seems to me .. and because it are pretty, yes large groups who receive the vaccination because of other reasons. It would be very unpleasant if you suddenly have a group of teachers who have to pay for vaccination, while the other gets it for free because of their asthma. (interview 6) |
| Motivation teachers not to vaccinate | But there was also quite a lot of resistance. That there were people who said “well why are you doing this?! What are you thinking?”. So they found it very patronizing and “what is the government interfering with”, well just to put it like that. (interview 1) |
|  | Well, privately I have quite some people working in education and a part of them really got angry because of the fact it was offered. Like “wow, is that how they want to reduce the absenteeism, this is not the way and I can decide for myself whether I want to be vaccinated or not.  But we did not systematically evaluate among teachers why they did not attend. (interview 2) |
|  | Uhm .. well let’s put it this way, and that is my own interpretation because I haven’t spoken to any teacher about what they think. But logistically it all started pretty late. I don’t know to what extent it was all known among teachers, and also to what extent the benefits were recognized by teachers. Uhm and also it is done as sort of support, making up for the teacher shortage. I can imagine, but I don’t know, that the feeling among teachers has been, like rather you buy something with the money, buy teachers for it or train teachers instead of offering a vaccine. I can imagine that, but really I have no if that feeling really was there. And I can also imagine that many teachers thought, but I’m young, what is the use of getting a vaccine as I am not part of a risk group. At least the considerations could have been taken into account .. maybe purely logistically “I cannot park my car”. (interview 4) |
|  | Well you know, people who are never ill and young people, why would they get a flu shot, you know. I understand very well that you do not always feel like it, and of course you have all those wild west stories that they can make you ill and things like that. (interview 5) |
| Motivation for teachers to vaccinate | Well probably onder het mom van every little thing helps. Or maybe I become a little older myself or I have a partner in my family or older parents that I can protect by doing this. (interview 4) |
|  | Teachers always have a great sense of responsibility and if there is a way to get ill less often or .. infect others, I think that quite a few are willing to do that. (interview 6) |
|  | I think the main reasons for teachers are for example the risk of getting ill is really decreasing then. Because teachers .. despite the image that might have emerged in the media lately, find it terrible to have to abandon their class. Teachers don’t want to get ill, no one ones to get ill. So the .. how do you say that .. as reason for the vaccination the significant reduction of the chance of getting ill in the flu season is a good incentive for a lot of teachers. (interview 7) |
| Disadvantages teacher influenza vaccination | That is also the reason why people often say “yes then you receive it, and I still get ill”. And then you don’t get ill from influenza, then you get ill from something else. In the winter, when influenza vaccines are offered, there are more things in the air (?). Then there is also the rhinovirus, the adenovirus, all winter-viruses pass by and people confuse those. And .. but it may be true that in the following week they are .. until the following week they are more susceptible to other viruses as the immune system is trying really hard to overcome the influenza virus .. or yes to overcome … to be ready for it when the virus comes along. (interview 9) |
| Advantages teacher influenza vaccination | Uhm well I work at schools where sometimes very, physically week children go. And I think that, on the one hand, it is of course pleasant for the teachers that they do not get ill. But also that they don’t infect others. And I think that children and parents would find it very pleasant that, in that way, they are in a safe environment where they cannot be infected.  But I think the advantages will differ per branch. [unintelligible] For one it is the absenteeism and for the other the much smaller risk of infection. (interview 6) |
|  | Well you know, of course, you’re hoping that .. that you get some sort of immune … well, of course we’ve heard the word “herd immunity” a few times recently. So that in itself would seem like a goal to me. Look, people go get that shot to prevent getting ill. That, of course, is their personal goal. The goal for me would be to make sure that I don’t get a shortage of staff, that is already [unintelligible] other level of abstraction. And finally, I think that on societal level we would like to see that herd immunity for a certain flu vaccine and thus protect each other too. (interview 7) |
|  | Uhm .. well yeah, I think you can say that the expectation is that there is simply less absenteeism. Especially in with shortages, every docent that drops out because of the flu is one too many. And well, if someone just has antibodies than you prevent them from infecting others , so it is not just that one teacher, but maybe also the danger of infection among teachers. And maybe also towards the target group, so the teacher with whom they, for who they are in front of the classroom who may be infected too. (interview 9) |
| Scientific research | Well if there is thorough research, that it would indeed be a very good idea to vaccinate teachers, then of course. But then it first must be properly scientifically studied. (interview 2) |
|  | If it turns out to be a very meaningful [unintelligible], I would heartily support that. I mean, it is only a small effort if it .. if certain goals are achieved then. Only then I do think we can make an effort how is it exactly useful. What should we end up with and to what extent do we reach that. So I’m not explicitly against, but it does have to serve a purpose, a goal. (interview 7) |
|  | Yes, then I would like to dive into the data myself first, like what are we talking about. How many people on average get ill if not vaccinated, and how many still get ill even though they are vaccinated .. and what is the effect on children of this. I would not like this … then you first really have to have done research to be able to form an opinion about it. (interview 8) |
| National policy | Or there must come some sort of advice from the health council, like teachers become a risk group for influenza, but then it’s something completely different. Then of course you are welcome and then we will start thinking about how to address this properly. (interview 2) |
|  | And from the city .. look, schoolboards are not under control of the stadsbestuur, so the city cannot decide on this. Then it would have to be on national level. Well I don’t really see that decision coming anytime soon. (interview 1) |
|  | Yes, my preference would then be that the RIVM or government assigns it as “this is important”, because every drop out in the educational sector costs the government money in the form of relief teachers of course, what eventually is paid by the board, but also a frustration of parents because children are kept at home that day or receive less education than usual. Then you recognize it as a national interest and then I think that this should simply be arranged by the [unintelligible] government. (interview 7) |

***References***

1. Elo S, Kyngas H. The qualitative content analysis process. J Adv Nurs. 2008;62(1):107-15.

2. Free flue vaccine for WSCC maintained school staff. Chichester: West sussex county council; 2018.

3. Novak L. No free flu jab for teachers. News. 2009 20 October 2009.

4. Clinical update: 2020 seasonal influenza vaccines - early advice for vaccination providers: Australian Government; [updated 2 March 2020]. Available from: <https://www.health.gov.au/news/clinical-update-2020-seasonal-influenza-vaccines-early-advice-for-vaccination-providers>.

5. Flu vaccine overview: National Health Service; 2019 [updated 16 July 2019]. Available from: <https://www.nhs.uk/conditions/vaccinations/flu-influenza-vaccine/>.
