# Supplementary material for: Influenza vaccination of school teachers: A scoping review and an impact estimation
Source: PLoS One. 2022 Aug 11;17(8):e0272332. doi: 10.1371/journal.pone.0272332 (PMC9371289; doi:10.1371/journal.pone.0272332)
Supplement: S4 File — (DOCX) [file pone.0272332.s004.docx]

**S4 Supplementary. Newspaper monitoring**

This supplement provides the following sections:

***Methods, Results, Tables and figures***

***Methods***

*Search terms used in news article database.* The LexisNexis database was used to identify Dutch news articles on teacher vaccination. These included articles from the Netherlands and from the Dutch speaking (Flemish) part of Belgium (January 2010 - 31 December 2019). Articles published in the first months of 2020 were not considered, as many were about the COVID-19 pandemic. Search terms were based on explorative searches. Three groups of search terms were established, containing terms related to influenza, vaccination or teachers (table S4.1 below). Search terms within each group were linked with Boolean operator “OR” and groups were linked together using Boolean operator “AND”, leading to results including at least one of the search terms in each subgroup. Dutch language (national and regional) newspapers were searched.

*Selecting news articles, charting and summarizing results.* All news articles were screened by title and full-text by one reviewer (AH). Subsequently, full-text assessment of the relevant news articles was conducted by one reviewer (AH). Characteristics recorded for all identified relevant articles were: date of publication, newspaper, newspaper region, article type, mentioned region and sentiment of the article. Both newspaper regions and mentioned regions were recorded specifically, but also subdivided by province and by larger categories of North/South/East/West of the Netherlands with North of the Netherlands including Groningen, Friesland and Drenthe; East of the Netherlands including Gelderland, Overijssel and Flevoland; South of the Netherlands including Limburg and Noord-Brabant; West of the Netherlands including Zeeland, Zuid-Holland, Noord-Holland and Utrecht. The same information was sometimes duplicately reported by different (electronic) newspapers. Therefore data was analysed twice: including duplicate coverage and excluding duplicate coverage. When excluding duplicates, the article was included in the data only once and newspaper region was then registered as “multiple” if the article was published in newspapers from different regions. If the news item was only published multiple times in different newspapers from the same region, then the specific region was recorded instead of “multiple”. If the news item was published in one specific region and was picked up by a national newspaper, containing mainly information concerning the specific region, then the specific region was recorded instead of “multiple”. Positive sentiment was noted when a positive opinion was expressed, or when the article described actual implementation of vaccination of school teachers. A negative opinion or non-implementation were considered as a negative sentiment. When articles did not have a clear overall sentiment because a combination of positive and negative opinions was expressed in the article, we considered the sentiment “multiple”.

***Results***

The Dutch newspaper search resulted in 243 hits of which 106 were identified as relevant. These 106 included 56 unique articles (table S4.2, below). The first was published in February 2015, describing problems schools faced in arranging substitute teachers. The next reports followed two years later in a Belgian newspaper, describing a lack of teachers and a role for influenza vaccination. From February 2018 onwards hits in Dutch newspaper increased up to 22, 21 and 10 in season-years 2017/2018, 2018/2019 and 2019/2020 respectively (figure S4.1, below). All articles were published during or just before the influenza seasons (September-March, figure S4.1, below), with three large peaks in February 2018 (n=20), November 2018 (n=14) and November 2019 (n=9), together 43 of the articles (76.8%, duplicates excluded). The majority of the articles were news articles (n=37, 66.1%). The other newspaper publications were opinion (n=8), column (n=7), interview (n=3) or readers letter (n=1) (table S4.3, below).

*Region of publication.* Thirty-two (30.2%) articles were published in a national newspaper (table S4.4, below). The remaining articles were published regionally in: Zuid-Holland (n=14), Noord-Holland (n=12), Zeeland (n=9), Utrecht (n=4), Noord-Brabant (n=6), Overijssel (n=5), and Gelderland (n=2) (figure S4.2 below). Five were published abroad (Belgium). Additionally, 17 (16.0%) news articles appeared in a local or regional newspaper that was distributed in multiple provinces, these provinces included Noord-Brabant (n=6), Zeeland (n=6), Noord-Holland (n=5), Zuid-Holland (n=3), Overijssel (n=3), Flevoland (n=3), Drenthe (n=3), Groningen (n=3) and Utrecht (n=2), leaving 2 provinces with no published articles detected (Friesland, Limburg). Combining these provinces in a northern, eastern, southern and western part of the Netherlands showed that most attention to the subject was given in the West (n=44) compared to East (n=10), South (n=7) and North (n=3) (table S4.5 and figure S4.1C, below).

*Region described.* The largest group of articles did not describe a specific region (n=20, 35.7%, excluding duplicates) (table S4.4, below). The remaining articles (n=36, 64.2%) described multiple provinces (n=10), or single provinces: Noord-Holland (n=8), Zeeland (n=5), foreign countries (n=3), Overijssel (n=3), Zuid-Holland (n=3), Noord-Brabant (n=2), Gelderland (n=1), Drenthe (n=1) (figure S4.2, below). Combining these provinces resulted in a similar distribution as for the newspaper region with the most attention for West (n=17), then East (n=4), South (n=2) and North (n=1). Of the 21 articles being published in a specific region and describing a specific region, 16 (75.2%) described the region the article is published in. The other 5 described their own region together with Overijssel (n=1) or together with a schoolboard with multiple locations in the country (n=4).

Eight schoolboards were described offering influenza vaccination to school teachers, located in Noord-Brabant (n=2, Roosendaal and Breda) (table S4.2 below, article 52), Overijssel (n=2, Twente and Hoonhorst) (table S4.2 below, article 30 and 36), Zeeland (n=3, Walcheren, Zeeuws-Vlaanderen and Noord- and Zuid-Beveland) (table S4.2 below, article 3 and 23) and a schoolboard that has different locations in the country (table S4.2 below, article 13, 16, 18, 22, 23 26). The latter stopped offering teacher vaccination as absenteeism reduced too little. However, details on the magnitude of reduction and how and where vaccination was offered by these boards are not given. Two boards considered vaccination (Assen, Drenthe and The Hague, Zuid-Holland) (table S4.2 below, article 28 and 15).and two decided not to implement. (Emmen, Drenthe and a schoolboard with different locations in the country) (table S4.2 below, article 28 and 40). A few arguments were described: questionable impact of influenza vaccination, privacy issues and organizational difficulties.

The overall sentiment of the articles was mainly positive (n=30, 53.6%, excluding duplicates) about vaccinating school teachers for influenza while 26.8% had no clear sentiment and 17.9% were negative (unknown: 1.8%) (figure S4.1B, below). In all seasons more positive than negative articles were published. Only in season 2018/2019 the number of positive and negative articles were almost comparable (table S4.6, below). Different parties expressed their opinion (table S4.6 and S4.7, below). Virologists were mainly positive (21 out of 23 articles), but the opinion of one virologist was described in many articles. Other health professionals, namely a psychologist and occupational physician were asked for their opinion in one of the articles, which was negative for the former and positive for the latter. School managements and schoolboards were mainly reported with positive views about vaccinating school teachers (n=6 for management, n=9 for board), although some also expressed negative opinions (n=1, n=5 respectively). Reported teachers views were positive (n=5) and negative (n=3). Whereas the primary and secondary school council (‘PO- and VO-raad’) were only positive in articles (6 out of 6), teacher unions (2 out of 2) and columnists or journalists (9 out of 10, as one was unknown) only expressed negative opinions about vaccinating school teachers for influenza. A positive opinion was given by an education councillor in 8 articles, always reporting on the same councillor. A local policy officer expressed a negative opinion. The majority of readers opinions was negative (4 out of 6), while two expressed a positive opinion.

***Tables and figures***

Table S4.1. Complete list of Dutch search terms sorted by corresponding subgroup for new article monitoring.

| Influenza | Vaccination | Teachers |
| --- | --- | --- |
| Griep* | Vaccin* | Leerkracht* |
| Influenza* | Prik | Docent* |
| Seizoensgriep* | Griepprik | Leraar |
|  |  | Leraren |
|  |  | Juf* |
|  |  | Basisschoolleraar |
|  |  | Basisschoolleraren |
|  |  | Schooldocent* |
|  |  | Werknemers onderwijs |

The asterisk (*) was used as a wildcard to allow end-truncation. Front truncation was not available.

Table S4.2. Characteristics of all relevant news article hits excluding duplicates.

|  | **Title** | **Publication date** | **Publication month** | **Newspaper** | **Newspaper region** | **Newspaper region (province)** | **Newspaper region (north/south/ east/west)** | **Article type (news / opinion)** | **Dutch / foreign** | **Regional / multiregional** | **Mentioned region in the article** | **Mentioned region in the article (province)** | **Mentioned region in the article (north/ south/east/west)** | **Sentiment** | **Mentioned persons** |
| --- | --- | --- | --- | --- | --- | --- | --- | --- | --- | --- | --- | --- | --- | --- | --- |
| 1 | Griepprik | 30-11-2019 | nov-19 | De Gazet van Antwerpen | Antwerpen | - | - | Column | foreign | Regional | Antwerpen | - | - | negative | columnist |
| 2 | Inktvlek | 30-11-2019 | nov-19 | Multiple | National | National | National | Column | Nederland |  | General | General | General | Unknown | columnist |
| 3 | Het is hoog tijd voor de griepprik | 9-11-2019 | nov-19 | Multiple | Zeeland | Zeeland | West | News | Nederland | Regional | Noord- en Zuid-Beveland + Zeeuws-Vlaanderen + Kapelle | Zeeland | West | Multiple: positive and negative | School board Noord- Zuid-Beveland (positive), schoolboard high school Zeeuws-Vlaanderen (negative, they stop offering it), school management Kapelle (negative, but stop offering it) |
| 4 | Griepprik voor Amsterdamse leraren ook in 2020 | 8-11-2019 | nov-19 | Multiple | Amsterdam | Noord-Holland | West | News | Nederland | Regional | Amsterdam | Noord-Holland | West | positive | Education councillor |
| 5 | Gratis griepprik voor leraren | 2-11-2019 | nov-19 | Trouw | National | National | National | News | Nederland | Regional | Amsterdam | Noord-Holland | West | positive | Education councillor |
| 6 | Leraar haalt gratis griepprik | 2-11-2019 | nov-19 | multiple | multiple | multiple | multiple | News | Nederland | Regional | Amsterdam | Noord-Holland | West | positive | Education councillor |
| 7 | Geen kop; Rechtersploeg MH17-proces compleet | 2-11-2019 | nov-19 | Nederlands Dagblad | National | National | National | News | Nederland | Regional | Amsterdam | Noord-Holland | West | positive | Education councillor |
| 8 | Kort nieuws Amsterdam | 2-11-2019 | nov-19 | Het Parool | Amsterdam | Noord-Holland | West | News | Nederland | Regional | Amsterdam | Noord-Holland | West | positive | Education councillor |
| 9 | Gratis griepprik voor Amsterdamse leraren | 1-11-2019 | nov-19 | multiple | Amsterdam | Noord-Holland | West | News | Nederland | multiregional | Amsterdam + Twente | Noord-Holland + Overijssel | West + East | positive | Education councillor |
| 10 | Onbegrijpelijk dat de onderwjiscrisis niet elke dag voorpaginanieuws is | 12-9-2019 | sep-19 | multiple | multiple | multiple | multiple | Interview | Nederland | Regional | Amsterdam | Noord-Holland | West | positive | Education councillor |
| 11 | Bied leraar griepprik aan | 8-2-2019 | feb-19 | AD/Haagsche Courant | Den Haag | Zuid-Holland | West | News | Nederland | Regional | Den Haag | Zuid-Holland | West | Positive | Political party (Christian Democratic Appeal) |
| 12 | Aantal griepprikken onder personeel van Adrz is verdubbeld | 29-1-2019 | jan-19 | multiple | multiple | multiple | multiple | News | Nederland | Regional | Kapelle | Zeeland | West | Positive | Schoolboard |
| 13 | Bonus voor griepprik verleidt docenten | 28-11-2018 | nov-18 | multiple | Zeeland | Zeeland | West | News | Nederland | multiregional | Amsterdam + Kapelle + Geldermalsen + Hardegarijp + Deventer + Hengelo + Hoorn + Utrecht | multiple | Multiple | Positive | Teachers + school management |
| 14 | Scholen nemen maatregelen tegen te verwachten griepgolf | 13-11-2018 | nov-18 | AD/Groene Hart | Randstad | Noord-Holland + Zuid-Holland + Utrecht | West | News | Nederland | multiregional | Goudse regio + Bodegraven-Reeuwijk + Gouda + Waddinxveen + Woerden + Alphen | Zuid-Holland + Utrecht | West | Multiple: positive and negative | Teachers (positive + negative) + school management (negative, but willing to adjust policy if it works well) + schoolboard (negative) |
| 15 | Griepprik leerkracht faciliteren | 13-11-2018 | nov-18 | AD/Haagsche Courant | Den Haag | Zuid-Holland | West | News | Nederland | Regional | Haaglanden + Haagse regio | Zuid-Holland | West | Multiple: positive and negative | Schoolboard (one positive, one negative) |
| 16 | Drukmiddel | 8-11-2018 | nov-18 | De Twentsche Courant Tubantia | Twente | Overijssel | East | Opinion | Nederland | multiregional | Twente + Amsterdam + Kapelle + Geldermalsen + Hardegarijp + Deventer + Hengelo + Hoorn + Utrecht | multiple | multiple | negative | Journalist |
| 17 | Snotteren | 8-11-2018 | nov-18 | De Twentsche Courant Tubantia | Twente | Overijssel | East | Opinion | Nederland | regional | Twente | Overijssel | East | negative | Journalist |
| 18 | Geachte redactie | 6-11-2018 | nov-18 | De Volkskrant | National | National | National | Opinion | Nederland | multiregional | Amsterdam + Kapelle + Geldermalsen + Hardegarijp + Deventer + Hengelo + Hoorn + Utrecht | multiple | multiple | positive | Schoolboard |
| 19 | Griepprik | 5-11-2018 | nov-18 | De Gelderlander | Gelderland | Gelderland | East | Column | Nederland |  | General | General | General | negative | Columnist |
| 20 | Gratis griepprik voor leraren: mogen zij wel ziek zijn? | 4-11-2018 | nov-18 | multiple | Amsterdam | Noord-Holland | West | News | Nederland | multiregional | Amsterdam + Kapelle | Noord-Holland + Zeeland | West | Multiple: positive and negative | Occupational physician /epidemiologist (positive) + psychologist (negative) |
| 21 | Griepprik | 3-11-2018 | nov-18 | De Volkskrant | National | National | National | Column | Nederland | Regional | Amsterdam | Noord-Holland | West | negative | Columnist |
| 22 | Worst | 3-11-2018 | nov-18 | De Volkskrant | National | National | National | Column | Nederland | multiregional | Amsterdam + Kapelle + Geldermalsen + Hardegarijp + Deventer + Hengelo + Hoorn + Utrecht | multiple | Multiple | negative | Columnist |
| 23 | Bonus als pleister op de prikplek | 2-11-2018 | nov-18 | multiple | Zeeland | Zeeland | West | News | Nederland | multiregional | Amsterdam + Kapelle + Geldermalsen + Hardegarijp + Deventer + Hengelo + Hoorn + Utrecht + Walcheren + Zeeuws-Vlaanderen | multiple | multiple | positive | Teachers + schoolboard |
| 24 | Inentbonus is ongepast | 2-11-2018 | nov-18 | Provinciale Zeeuwse Courant | Zeeland | Zeeland | West | Opinion | Nederland | Regional | Kapelle | Zeeland | West | negative | Readers |
| 25 | Dat geld is een leuke bijkomstigheid | 2-11-2018 | nov-18 | Provinciale Zeeuwse Courant | Zeeland | Zeeland | West | News | Nederland | Regional | Kapelle | Zeeland | West | positive | Teachers |
| 26 | Scholen bestrijden griep met gratis vaccin en 405 euro | 1-11-2018 | nov-18 | De Volkskrant | National | National | National | News | Nederland | multiregional | Amsterdam + Kapelle + Geldermalsen + Hardegarijp + Deventer + Hengelo + Hoorn + Utrecht + Twente | multiple | multiple | Positive | Virologist + schoolboard + PO-raad (primary education council) |
| 27 | Gratis griepprik voor leraren | 31-10-2018 | oct-18 | multiple | Amsterdam | Noord-Holland | West | News | Nederland | Regional | Amsterdam | Noord-Holland | West | positive | Education councillor |
| 28 | Scholen zien griep met angst en beven komen | 15-10-2018 | oct-18 | Dagblad van het Noorden | Groningen + Drenthe | Groningen + Drenthe | North | News | Nederland | Regional | Emmen + Assen | Drenthe | North | Multiple: positive and negative | Schoolboard (positive) + policy officer (negative) |
| 29 | Prik | 12-10-2018 | oct-18 | multiple | Noordoosten en midden Noord-Brabant | Noord-Brabant | South | Column | Nederland |  | General | General | General | negative | columnist |
| 30 | Gratis griepprik voor docenten in Twents onderwijs | 27-9-2018 | sep-18 | De Twentsche Courant Tubantia | Twente | Overijssel | East | News | Nederland | Regional | Twente | Overijssel | East | positive | Schoolboard (positive) + virologist (positive) |
| 31 | Alle leerkrachten een griepprik? | 19-9-2018 | sep-18 | De Twentsche Courant Tubantia | Twente | Overijssel | East | Opinion | Nederland |  | General | General | General | positive | Readers |
| 32 | Hopen dat de geest helder blijft | 22-3-2018 | mar-18 | AD/Rotterdams Dagblad | Rotterdam | Zuid-Holland | West | Interview | Nederland |  | General | General | General | positive | Virologist |
| 33 | Griep | 3-3-3018 | mar-18 | Reformatorisch Dagblad | National | National | National | News | Nederland |  | General | General | General | Multiple: positive and negative | Virologist (positive) + journalist (negative) |
| 34 | Van wie komt die claim? | 13-2-2018 | feb-18 | De Volkskrant | National | National | National | Interview | Nederland |  | General | General | General | Multiple: positive and negative | Virologist (one positive, one negative) |
| 35 | Juf is ziek, maar daar is de oud-juf | 10-2-2018 | feb-18 | multiple | National | National | National | News | Nederland | Regional | Vught | Noord-Brabant | South | Multiple: positive and negative | Virologist (positive) + teachers (negative) + school management (positive) |
| 36 | Dagboek van een schooldirecteur in grieptijd | 10-2-2018 | feb-18 | multiple | multiple | multiple | multiple | News | Nederland | Regional | Hoonhorst + Heino | Overijssel | East | neutral | School management |
| 37 | Griepprik | 9-2-2018 | feb-18 | multiple | Noordoosten en midden Noord-Brabant | Noord-Brabant | South | Column | Nederland |  | General | General | General | Multiple: positive and negative | Columnist (negative) + virologist (positive) |
| 38 | Waarom ik (65) voorlopig toch maar geen griepprik ga halen | 8-2-2018 | feb-18 | multiple | multiple | multiple | multiple | Letter | Nederland |  | General | General | General | Negative | Readers |
| 39 | De klas naar huis, het ziekenhuis vol | 8-2-2018 | feb-18 | Reformatorisch Dagblad | National | National | National | News | Nederland |  | General | General | General | positive | Virologist + PO-raad (primary education council) |
| 40 | Met een griepprik voor leerkrachten ben je er niet | 7-2-2018 | feb-18 | Nederlands Dagblad | National | National | National | News | Nederland | Multiregional | Doorn + Driebergen + Wijk bij Duurstede + Vereniging Gereformeerd Onderwijs (veel locaties) | multiple | multiple | Multiple: positive and negative | Virologist (positive) + schoolboard VGS (negative) + VO-raad (secondary education council, positive) |
| 41 | School wil geen prik voor de juf | 7-2-2018 | feb-18 | multiple | Rotterdam | Zuid-Holland | West | News | Nederland | Regional | Rotterdam | Zuid-Holland | West | Multiple: positive and negative | Virologist (positive) + schoolboard (negative) + teachers (one positive, one negative) |
| 42 | Leraar beslist zelf over de griepprik | 7-2-2018 | feb-18 | Metro | National | National | National | News | Nederland |  | General | General | General | Multiple: positive and negative | Teacher union (negative) + virologist (positive) + PO-raad (primary education council, positive) |
| 43 | Twitter | 7-2-2018 | feb-18 | Provinciale Zeeuwse Courant | Zeeland | Zeeland | West | Opinion (tweet) | Nederland |  | General | General | General | negative | Readers |
| 44 | Viroloog van Ranst vindt griepprik voor leerkrachten overbodig | 7-2-2018 | feb-18 | Het Belang van Limburg | Belgisch-Limburg | - | - | News | foreign |  | General | General | General | Multiple: positive and negative | Virologist (one positive, one negative) |
| 45 | Geachte lezer | 7-2-2018 | feb-18 | De Telegraaf | National | National | National | Opinion | Nederland |  | General | General | General | Multiple: positive and negative | Readers (one positive, one negative) |
| 46 | Iedereen verplichte griepprik | 7-2-2018 | feb-18 | multiple | multiple | multiple | multiple | Opinion | Nederland |  | General | General | General | positive | Virologist (positive) |
| 47 | Griepvrij voor scholier | 6-2-2018 | feb-18 | De Telegraaf | National | National | National | News | Nederland | Regional | Zeeland | Zeeland | West | positive | Virologist (positive) + school management (positive) + PO-raad (primary education council, positive) |
| 48 | Griepprik geen verplichting voor leraar | 6-2-2018 | feb-18 | Algemeen Nederlands Persbureau | National | National | National | News | Nederland |  | General | General | General | Multiple: positive and negative | Teacher union (negative) + virologist (positive) + PO-raad (primary education council, positive) |
| 49 | Expert Osterhaus: geef alle leraren griepprik | 6-2-2018 | feb-18 | Algemeen Nederlands Persbureau | National | National | National | News | Nederland |  | General | General | General | positive | Virologist |
| 50 | Viroloog wil voor alle leraren een griepprik | 6-2-2018 | feb-18 | Reformatorisch Dagblad | National | National | National | News | Nederland |  | General | General | General | positive | Virologist |
| 51 | Griepprik redding onderwijs | 6-2-2018 | feb-18 | De Telegraaf | National | National | National | News | Nederland |  | General | General | General | positive | Virologist |
| 52 | Grieperige juf kan beter thuisblijven, adviseert arts | 6-2-2018 | feb-18 | BN/DeStem | West Noord-Brabant / Zeeland | Noord-Brabant + Zeeland | South + West | News | Nederland | Regional | Roosendaal | Noord-Brabant | South | positive | Schoolboard |
| 53 | Schooldirecties moeten griepprik aanbieden | 6-2-2018 | feb-18 | BNR nieuwsradio | National | National | National | News | Nederland |  | General | General | General | positive | Virologist |
| 54 | Viroloog wil leraren vaccineren tegen de griep | 31-1-2017 | jan-17 | multiple | multiple | - | - | News | foreign | Regional | Antwerpen | - | - | positive | Virologist |
| 55 | Viroloog Marc van Ranst: " Vaccineer leerkrachten" | 31-1-2017 | jan-17 | De Gazet van Antwerpen | Antwerpen | - | - | News | foreign | Regional | Antwerpen | - | - | positive | Virologist |
| 56 | Scholen puffend naar de meet | 20-2-2015 | feb-15 | De Stentor | Flevoland + Achterhoek +Noordwest-Overijssel + Veluwe + Salland | Flevoland + Overijssel | East | News | Nederland | Regional | Apeldoorn | Gelderland | East | positive | School management |

Fig S4.1. Number of articles published according to publication date (excluding duplicates).


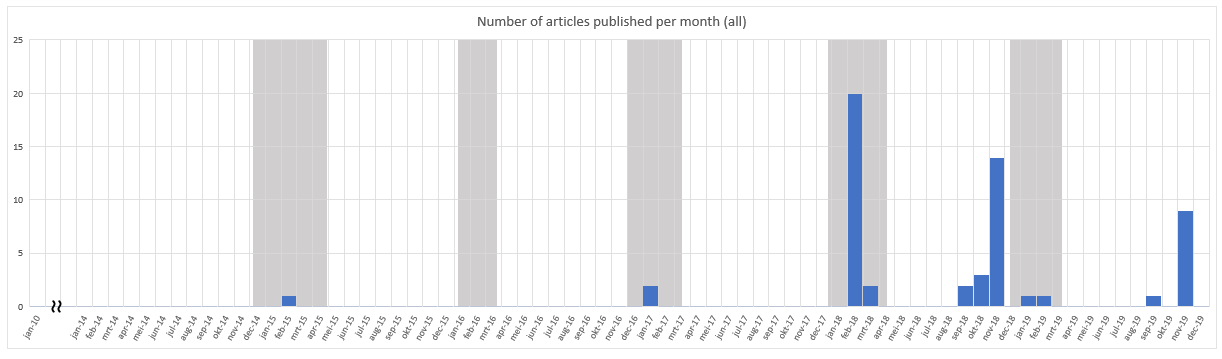
1A. Number of articles published per month


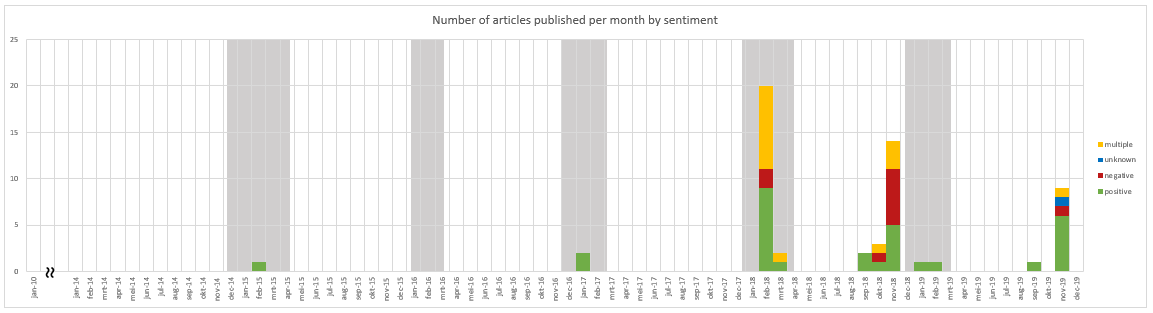
1B. Number of articles published per month by sentiment

1C. Number of articles published per month by mentioned region (west versus other)


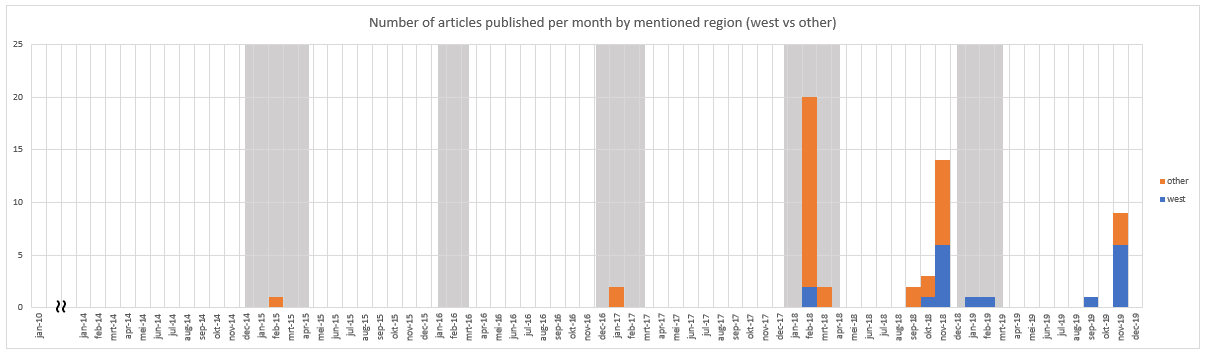


1D. Number of articles published per month by published region (west vs other)


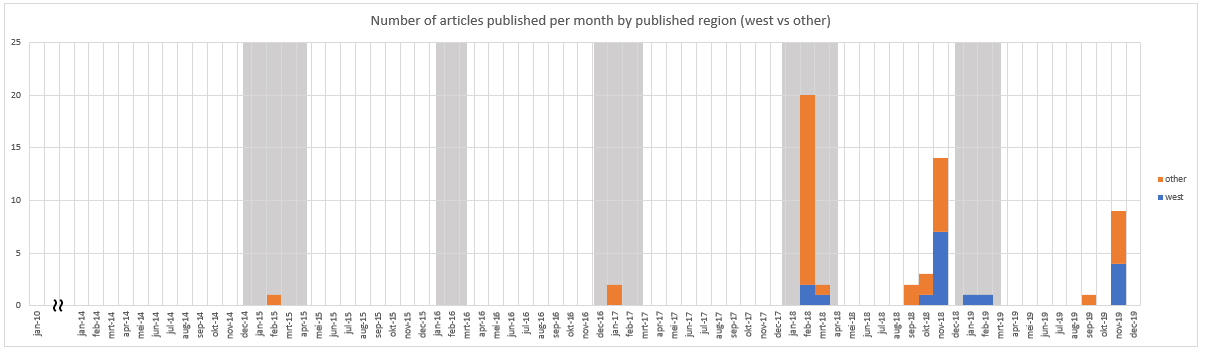


Grey areas highlight the influenza epidemics in the Netherlands. Yellow = multiple sentiments, bleu = unknown sentiment, red = negative sentiment, green = positive sentiment.

Table S4.3. Type of articles (excluding duplicates)

| **Sort of article** | **Number** |
| --- | --- |
| Opinion | 8 |
| News | 37 |
| Column | 7 |
| Interview | 3 |
| Readers letter | 1 |
| Total | 56 |

Table S4.4. Number of articles per newspaper region and mentioned region.

| REGION | Number of articles per newspaper region (including duplicates) | Number of articles per newspaper region (excluding duplicates | Number of articles per mentioned region (excluding duplicates) |
| --- | --- | --- | --- |
| Noord-Brabant | 6 | 2 | 2 |
| Limburg | 0 | 0 | 0 |
| Zeeland | 9 | 7 | 5 |
| Zuid-Holland | 14 | 4 | 3 |
| Noord-Holland | 12 | 5 | 8 |
| Utrecht | 4 | 0 | 0 |
| Gelderland | 2 | 1 | 1 |
| Overijssel | 5 | 4 | 3 |
| Flevoland | 0 | 0 | 0 |
| Drenthe | 0 | 0 | 1 |
| Groningen | 0 | 0 | 0 |
| Friesland | 0 | 0 | 0 |
| National | 32 | 20 | 0 |
| Foreign | 5 | 4 | 3 |
| Multiple provinces | **17** | **9** | **10** |
| Noord-Brabant | 6 | 1 | 0 |
| Limburg | 0 | 0 | 0 |
| Zeeland | 6 | 1 | 3 |
| Zuid-Holland | 3 | 1 | 1 |
| Noord-Holland | 5 | 1 | 7 |
| Utrecht | 2 | 1 | 4 |
| Gelderland | 0 | 0 | 2 |
| Overijssel | 3 | 1 | 6 |
| Flevoland | 3 | 1 | 0 |
| Drenthe | 3 | 1 | 0 |
| Groningen | 3 | 1 | 0 |
| Friesland |  |  | 2 |
| General* |  |  | 20 |

* General: no specific region was mentioned in the article

Table S4.5. Number of articles per newspaper region and mentioned region (north/east/south/west).

| REGION | Number of articles per newspaper region (including duplicates) | Number of articles per newspaper region (excluding duplicates | Number of articles per mentioned region (excluding duplicates) |
| --- | --- | --- | --- |
| North | 3 | 1 | 1 |
| East | 10 | 6 | 4 |
| South | 7 | 2 | 2 |
| West | 44 | 17 | 18 |
| National | 32 | 20 | 0 |
| Foreign | 5 | 4 | 3 |
| Multiple | **5** | **6** | **8** |
| North |  |  |  |
| East |  |  | 1 |
| South | 5 | 1 |  |
| West | 5 | 1 | 1 |
| General* |  |  | 20 |

* General: no specific region was mentioned in the article

Table S4.6. Sentiment of the articles (excluding duplicates).

| SENTIMENT | number | percentage of total | 2014-2017 | 2017/2018 | 2018/2019 | 2019/2020 |
| --- | --- | --- | --- | --- | --- | --- |
| Positive | 30 | 53.6 | 3 | 13 | 10 | 7 |
| Negative | 10 | 17.9 | 0 | 2 | 7 | 1 |
| Unknown | 1 | 1.8 | 0 | 0 | 0 | 1 |
| Multiple (positive and negative) | 15 | 26.8 | 0 | 10 | 4 | 1 |
| TOTAL | **56** |  | **3** | **22** | **21** | **10** |

Table S4.7. Sentiment of the articles per person mentioned in the article (excluding duplicates).

| **SENTIMENT PER PERSON** | **Positive** | **Negative** | **Unknown** |
| --- | --- | --- | --- |
| Virologist | 21 | 2 | 0 |
| School management | 6 | 1 | 0 |
| Schoolboard | 9 | 5 | 0 |
| Teachers | 5 | 3 | 0 |
| PE-council | 5 | 0 | 0 |
| SE-council | 1 | 0 | 0 |
| Teacher union | 0 | 2 | 0 |
| Reader | 2 | 4 | 0 |
| Columnist | 0 | 6 | 1 |
| Journalist | 0 | 3 | 0 |
| Education councillor | 8 | 0 | 0 |
| Policy officer | 0 | 1 | 0 |
| Psychologist | 0 | 1 | 0 |
| Occupational physician | 1 | 0 | 0 |

PE = primary education

SE = secondary education

Fig S4.2. Number of news articles published on influenza vaccination for teachers by province* (excluding duplicate coverage in multiple newspapers)


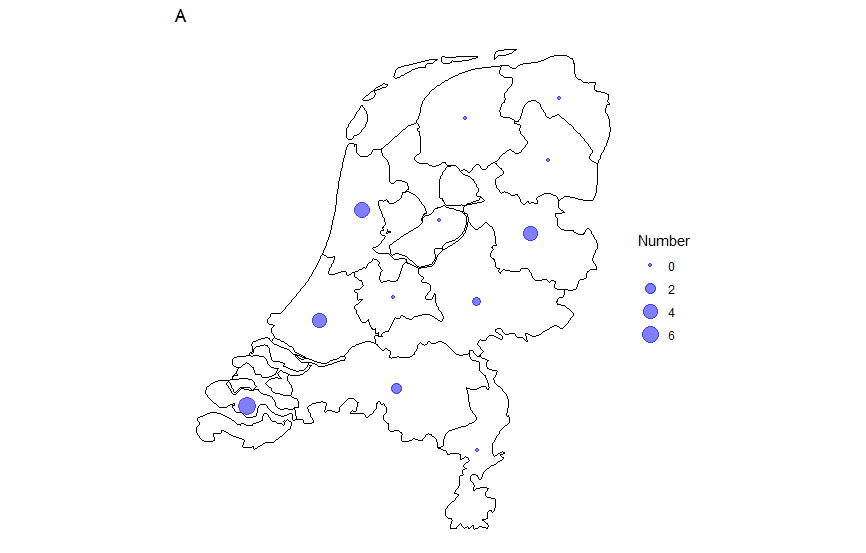

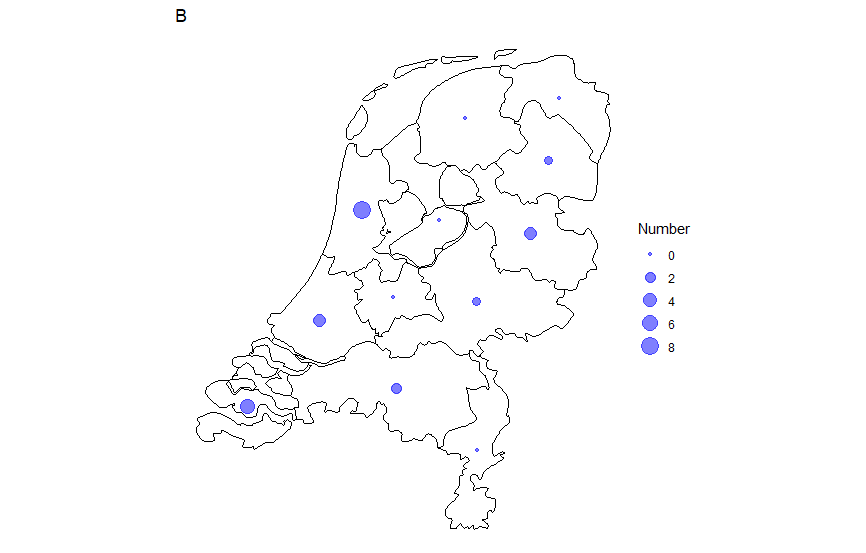


A: Number of newsarticles published on influenza vaccination for teachers by province (irrespective of the region mentioned in the article).

B: Number of newsarticles mentionings per specific province.

* province: data on province areas as available from Statistics Netherlands (CBS) and the Dutch land registry (Kadaster). Metadata are freely downloadable without copyright from the Dutch National Geo Registry: <https://www.nationaalgeoregister.nl/geonetwork/srv/dut/catalog.search#/metadata/effe1ab0-073d-437c-af13-df5c5e07d6cd?tab=general>). The figure is made using statistical software R [R Core Team (2021). R: A language and environment for statistical computing. R Foundation for Statistical Computing, Vienna, Austria. <https://www.R-project.org/>]
