# Supplementary material for: Influenza vaccination of school teachers: A scoping review and an impact estimation
Source: PLoS One. 2022 Aug 11;17(8):e0272332. doi: 10.1371/journal.pone.0272332 (PMC9371289; doi:10.1371/journal.pone.0272332)
Supplement: S5 File — (DOCX) [file pone.0272332.s005.docx]

**S5 Supplementary. Vaccine impact calculations**

This supplement provides figure 1 and tables 1-6

**Figure S5.1. Calculation of number of potentially averted influenza related events for Amsterdam**

| Also for Amsterdam potentially averted events were calculated per hypothetical vaccination uptake scenarios (scenario’s: 2%, 10%, 25%,50% and 70% uptake). However, for the 2% scenario we adjusted for the fact that Amsterdam already had a vaccination programme in place with a vaccination uptake of 2%. For this, we used the following formula (19, 20):  $Number of averted events=N-n= \frac{n}{1-\left( VU*VE \right)}-n=n*(\frac{VU*VE}{1-\left( VU*VE \right)})$  N = estimated total number of events without the vaccination programme, as calculated in the bullets 1 to 3 in figure 1 in the manuscript. This number is not known in the situation in Amsterdam where the vaccination campaign was already in place.  n = estimated total number of events with the vaccination programme, calculated similarly to N.  VU = vaccination uptake (of the in place vaccination programme)  VE = vaccine effectiveness |
| --- |

**Table S5.1. Estimated cases per event per season for the Netherlands.**

|  | 2016/2017 | 2017/2018 | 2018/2019 | 2019/2020 |
| --- | --- | --- | --- | --- |
| ESTIMATED EVENTS |  |  |  |  |
| Estimated notifications influenza absenteeism (per year) | **46,732** | **62,996** | **46,479** | n.a. |
| Estimated total days of influenza absenteeism (per year) | **141,262** | **207,886** | **162,594** | n.a. |
| Estimated total influenza GP visits (per year) | **1,292** | **2,111** | **1,079** | n.a. |

n.a. = not available

GP = general practitioner

**Table S5.2. Estimated number of cases per event and its input parameters for Amsterdam**

|  | 2016/2017 | 2017/2018 | 2018/2019 | 2019/2020 |
| --- | --- | --- | --- | --- |
| INPUT PARAMETERS |  |  |  |  |
| Teacher population PE^1^ | 5,651 | 5,591 | 5,610 | 5,532 |
| Teacher population SE^2^ | 3,777 | 3,766 | 3,787 | 3,859 |
| Absenteeism notifications PE (per teacher per year)^3^ | 1.35 | 1.22 | 1.37 | n.a. |
| Absenteeism notifications SE (per teacher per year)^3^ | 2.1 | 1.9 | 2.2 | n.a. |
| Average duration per absenteeism PE (days per absence)^3^ | 3.3 | 3.3 | 2.7 | n.a. |
| Average duration per absenteeism SE  (days per absence)^3^ | 2.8 | 3.3 | 3.6 | n.a. |
| Self-reported flu as reason for last absence (%)^4, 5, 6^ | 39.5 | 39.6 | 39.9 | n.a. |
| Influenza-like illness GP visits (age 15-44) (per year per 10,000)*^7^ | 134 | 171 | 109 | n.a. |
| Influenza-like illness GP visits (age 45-64) (per year per 10,000)*^7^ | 177 | 154 | 143 | n.a. |
| Average influenza-like illness GP visits (age 15-64) (per year per 10,000)* | 155.5 | 162.5 | 126 | n.a. |
| Rate of specimens testing positive for influenza from influenza-like illness GP visits (age 15-64)^7^ | 0.41 | 0.64 | 0.42 | n.a. |
| ESTIMATED EVENTS |  |  |  |  |
| Estimated notifications influenza absenteeism (per year) | **2,520** | **3,542** | **2,684** | n.a. |
| Estimated total days of influenza absenteeism (per year) | **7,674** | **11,689** | **8,504** | n.a. |
| Estimated total influenza GP visits (per year) | **60** | **97** | **50** | n.a. |

n.a. = not available

PE = primary education

SE = secondary education

GP = general practitioner

* = in respiratory season (week 40 – week 20)

^1^ Report DUO: onderwijspersoneel PO in personen 2011-2019 [1]; ^2^ Report DUO: onderwijspersoneel VO in personen 2011-2019 [2]; ^3^ Report DUO: verzuimkengetallen 2016-2018 [3]; ^4, 5, 6^ Report CBS: nationale enquête arbeidsomstandigheden 2016, 2017 and 2018 [4-6]; ^7^Report RIVM: annual surveillance of influenza and other respiratory infections in the Netherlands [7].

**Table S5.3. Estimated number of cases per event and its input parameters for Rotterdam**

|  | 2016/2017 | 2017/2018 | 2018/2019 | 2019/2020 |
| --- | --- | --- | --- | --- |
| INPUT PARAMETERS |  |  |  |  |
| Teacher population PE^1^ | 4,690 | 4,633 | 4,585 | 4,569 |
| Teacher population SE^2^ | 3,020 | 2,914 | 2,942 | 2,990 |
| Absenteeism notifications PE (per teacher per year)^3^ | 1.16 | 1.0 | 1.18 | n.a. |
| Absenteeism notifications SE (per teacher per year)^3^ | 1.7 | 1.6 | 1.7 | n.a. |
| Average duration per absenteeism PE (days per absence)^3^ | 3.0 | 2.6 | 2.8 | n.a. |
| Average duration per absenteeism SE  (days per absence)^3^ | 1.9 | 2.5 | 2.6 | n.a. |
| Self-reported flu as reason for last absence (%)^4, 5, 6^ | 39.5 | 39.6 | 39.9 | n.a. |
| Influenza-like illness GP visits (age 15-44) (per year per 10,000)*^7^ | 134 | 171 | 109 | n.a. |
| Influenza-like illness GP visits (age 45-64) (per year per 10,000)*^7^ | 177 | 154 | 143 | n.a. |
| Average influenza-like illness GP visits (age 15-64) (per year per 10,000)* | 155.5 | 162.5 | 126 | n.a. |
| Rate of specimens testing positive for influenza from influenza-like illness GP visits (age 15-64)^7^ | 0.41 | 0.64 | 0.42 | n.a. |
| ESTIMATED EVENTS |  |  |  |  |
| Estimated notifications influenza absenteeism (per year) | **1,713** | **2,356** | **1,745** | n.a. |
| Estimated total days of influenza absenteeism (per year) | **4,223** | **6,007** | **4,718** | n.a. |
| Estimated total influenza GP visits (per year) | **49** | **78** | **40** | n.a. |

n.a. = not available

PE = primary education

SE = secondary education

GP = general practitioner

* = in respiratory season (week 40 – week 20)

^1^ Report DUO: onderwijspersoneel PO in personen 2011-2019 [1]; ^2^ Report DUO: onderwijspersoneel VO in personen 2011-2019 [2]; ^3^ Report DUO: verzuimkengetallen 2016-2018 [3]; ^4, 5, 6^ Report CBS: nationale enquête arbeidsomstandigheden 2016, 2017 and 2018 [4-6]; ^7^Report RIVM: annual surveillance of influenza and other respiratory infections in the Netherlands [7].

**Table S5.4. Estimated number of averted influenza events for the Netherlands per season by different vaccine uptake scenarios.**

|  | Vaccine uptake (%) | 2016/2017 | 2017/2018 | 2018/2019 |
| --- | --- | --- | --- | --- |
| Estimated number of averted notifications influenza absenteeism* | **2** | 301 | 350 | 128 |
|  | **10** | 1507 | 1751 | 639 |
|  | **25** | 3768 | 4378 | 1598 |
|  | **50** | 7537 | 8756 | 3195 |
|  | **70** | 10552 | 12259 | 4474 |
| Estimated number of averted days of influenza absenteeism* | **2** | 911 | 1156 | 447 |
|  | **10** | 4557 | 5779 | 2236 |
|  | **25** | 11391 | 14448 | 5589 |
|  | **50** | 22783 | 28896 | 11178 |
|  | **70** | 31896 | 40455 | 15650 |
| Estimated number of averted influenza GP visits | **2** | 8 | 12 | 3 |
|  | **10** | 42 | 59 | 15 |
|  | **25** | 104 | 147 | 37 |
|  | **50** | 208 | 293 | 74 |
|  | **70** | 292 | 411 | 104 |

*self-reported flu as reason for absenteeism

**Table S5.5. Estimated number of averted influenza events for Amsterdam per season by different vaccine uptake scenarios.**

|  | Vaccine uptake (%) | 2016/2017 | 2017/2018 | 2018/2019 |
| --- | --- | --- | --- | --- |
| Estimated number of averted notifications influenza absenteeism* | 2 | 16 | 20 | 7** |
|  | 10 | 81 | 98 | 37 |
|  | 25 | 203 | 246 | 92 |
|  | 50 | 406 | 492 | 185 |
|  | 70 | 569 | 689 | 258 |
| Estimated number of averted days of influenza absenteeism* | 2 | 50 | 65 | 23** |
|  | 10 | 248 | 325 | 117 |
|  | 25 | 619 | 812 | 292 |
|  | 50 | 1238 | 1625 | 585 |
|  | 70 | 1733 | 2275 | 818 |
| Estimated number of averted influenza GP visits | 2 | 0 | 1 | 0** |
|  | 10 | 2 | 3 | 1 |
|  | 25 | 5 | 7 | 2 |
|  | 50 | 10 | 14 | 3 |
|  | 70 | 14 | 19 | 5 |

*self-reported flu as reason for absenteeism

**this was a true situation; teachers were offered influenza vaccination in Amsterdam in 2018/2019 and 2% received it.

**Table S5.6. Estimated number of averted influenza events for Rotterdam per season by different vaccine uptake scenarios.**

|  | Vaccine uptake (%) | 2016/2017 | 2017/2018 | 2018/2019 |
| --- | --- | --- | --- | --- |
| Estimated number of averted notifications influenza absenteeism* | 2 | 11 | 13 | 5 |
|  | 10 | 55 | 65 | 24 |
|  | 25 | 138 | 164 | 60 |
|  | 50 | 276 | 327 | 120 |
|  | 70 | 387 | 458 | 168 |
| Estimated number of averted days of influenza absenteeism* | 2 | 27 | 33 | 13 |
|  | 10 | 136 | 167 | 65 |
|  | 25 | 341 | 417 | 162 |
|  | 50 | 681 | 835 | 324 |
|  | 70 | 954 | 1169 | 454 |
| Estimated number of averted influenza GP visits | 2 | 0 | 0 | 0 |
|  | 10 | 2 | 2 | 1 |
|  | 52 | 4 | 5 | 1 |
|  | 50 | 8 | 11 | 3 |
|  | 70 | 11 | 15 | 4 |

*self-reported flu as reason for absenteeism

***References***

1. Educational staff numbers primary education 2011-2019 [in Dutch: Onderwijspersoneel po in personen 2011-2019]: Dienst Uitvoering Onderwijs; [updated 31 March 2020]. Available from: <https://duo.nl/open_onderwijsdata/databestanden/po/onderwijspersoneel/po-personeel1.jsp>.

2. Educational staff numbers secundary education 2011-2019 [in Dutch: Onderwijspersoneel vo in personen 2011-2019]: Dienst Uitvoering Onderwijs; [updated 31 March 2020]. Available from: <https://duo.nl/open_onderwijsdata/databestanden/vo/onderwijspersoneel/vo-personeel1.jsp>.

3. Absenteeism numbers of educational staff 2016-2018 [in Dutch: Verzuimkengetallen 2016-2018]: Dienst Uitvoering Onderwijs; 2019 [updated 6 September 2019]. Available from: <https://duo.nl/open_onderwijsdata/publicaties/verzuim-personeel-po-vo/verzuim-personeel.jsp>.

4. Hooftman W, Mars G, Janssen B, de Vroome E, Janssen B, Pleijers A, et al. National survey working conditions 2018 [in Dutch: Nationale Enquête Arbeidsomstandigheden 2018]. CBS, 2019.

5. Hooftman W, Mars G, Janssen B, de Vroome E, Janssen B, Ramaekers M, et al. National survey working conditions 2017 [in Dutch: Nationale Enquête Arbeidsomstandigheden 2017]. CBS, 2018.

6. Hooftman W, Mars G, Janssen B, de Vroome E, Michiels J, Pleijers A, et al. National survey working conditions 2016 [in Dutch: Nationale Enquête Arbeidsomstandigheden 2016]. CBS, 2017.

7. Reukers D, van Asten L, Brandsema P, Dijkstra F, Donker G, van Gageldonk-Lafeber A, et al. Annual report Surveillance of influenza and other respiratory infections in the Netherlands: winter 2018/2019. Rijksinstituut voor Volkgezondheid en Milieu RIVM, 2019. Report No.
