# Supplementary material for: Influenza vaccination of school teachers: A scoping review and an impact estimation
Source: PLoS One. 2022 Aug 11;17(8):e0272332. doi: 10.1371/journal.pone.0272332 (PMC9371289; doi:10.1371/journal.pone.0272332)
Supplement: Attachment — Submitted filename: Response to reviewers.docx [file pone.0272332.s007.docx]

We thank the reviewers for their comments and suggestions. We believe they have greatly contributed to improving the quality and clarity of the work. Below, we provide a point by point response to all their points and to the additional PLOS ONE requirements.

**PloS One additional requirements.**

1. Please ensure that your manuscript meets PLOS ONE's style requirements, including those for file naming.

**Reply:** Thank you, we have checked the file names and all other PLOS ONE requirements.

2. We note that you have indicated that data from this study are available upon request. PLOS only allows data to be available upon request if there are legal or ethical restrictions on sharing data publicly. For information on unacceptable data access restrictions, please see <http://journals.plos.org/plosone/s/data-availability#loc-unacceptable-data-access-restrictions>.

**REPLY:** Checking the provided PLOS ONE link we have now changed this. We have made the data available in the manuscript, the anonimized excerpts of the interviews are in supplement S3 (pages 10-18).

In your revised cover letter, please address the following prompts:

 a) If there are ethical or legal restrictions on sharing a de-identified data set, please explain them in detail (e.g., data contain potentially identifying or sensitive patient information) and who has imposed them (e.g., an ethics committee). Please also provide contact information for a data access committee, ethics committee, or other institutional body to which data requests may be sent.

**REPLY:** We checked our information letter and our consent form and we have now made the data available in the manuscript, see also point b below.

b) If there are no restrictions, please upload the minimal anonymized data set necessary to replicate your study findings as either Supporting Information files or to a stable, public repository and provide us with the relevant URLs, DOIs, or accession numbers. Please see <http://www.bmj.com/content/340/bmj.c181.long> for guidelines on how to de-identify and prepare clinical data for publication. For a list of acceptable repositories, please see <http://journals.plos.org/plosone/s/data-availability#loc-recommended-repositories>.

**REPLY:** We have added the anonymized excerpts of the interviews in supplementary S3 (pages 10-18).

We will update your Data Availability statement on your behalf to reflect the information you provide.

3. Our staff editors have determined that your manuscript is likely within the scope of our Call for Papers on Influenza. This editorial initiative is headed by PLOS ONE Guest Editors Dr. Meagan Deming and Dr. Deshayne Fell. The Collection encompasses research on influenza prevention on every level, including in vitro, translational, behavioral, and clinical studies; disease and immunity modelling; as well as new approaches to influenza prevention. Additional information can be found on our announcement page: <https://collections.plos.org/call-for-papers/influenza/>.

Currently, your manuscript is included in the group of papers being considered for this call. Please note that being considered for the Collection does not require additional peer review beyond the journal’s standard process and will not delay the publication of your manuscript if it is accepted by PLOS ONE. We would greatly appreciate your confirmation that you would like your manuscript to be considered for this Collection by indicating this in your next cover letter. If you would prefer to remove your manuscript from collection consideration, please specify this in your cover letter.

**REPLY:** we are certainly happy for publication within, or outside of this call, as PLOS ONE would wish.

4. Please provide additional details regarding participant consent. In the ethics statement in the Methods and online submission information, please ensure that you have specified whether consent was informed.

**REPLY:** We have added additional detail in the online submission form as well as to the methods section. For this we added the following sentences to supplement S3:

‘Potential participants were approached by email and they received a letter asking whether they would agree to participate. The letter explained the research aims, interview procedures, and privacy matters (de-identification of transcripts). Written and oral consent was obtained from each consenting interviewee. The consent form included queries on whether the interviewee had read the information letter and whether all their queries and concerns had been addressed satisfactorily. Ethical approval was not required for this study according to Dutch legislation, as it concerned a once only interview.’ (Supplement S3, page 1-2, lines 26-38)

5. We note that Figure 3 in your manuscript and Figure 2 in S4 file contain map images which may be copyrighted. All PLOS content is published under the Creative Commons Attribution License (CC BY 4.0), which means that the manuscript, images, and Supporting Information files will be freely available online, and any third party is permitted to access, download, copy, distribute, and use these materials in any way, even commercially, with proper attribution. For these reasons, we cannot publish previously copyrighted maps or satellite images created using proprietary data, such as Google software (Google Maps, Street View, and Earth). For more information, see our copyright guidelines: <http://journals.plos.org/plosone/s/licenses-and-copyright>.

**REPLY:** We have now produced these map images using the free R statistical package, which is without copyright *[R Core Team (2021). R: A language and environment for statistical computing. R Foundation for Statistical Computing, Vienna, Austria.* [*https://www.R-project.org/*](https://www.R-project.org/)*]*. The source data on province areas as available from Statistics Netherlands (CBS) and the Dutch land registry (Kadaster). Metadata are freely downloadable without copyright from the Dutch National Geo Registry: https://www.nationaalgeoregister.nl/geonetwork/srv/dut/catalog.search#/metadata/effe1ab0-073d-437c-af13-df5c5e07d6cd?tab=general).

To clarify these two sources for readers we have added the following subscript below the figure: ‘* province: data on province areas as available from Statistics Netherlands (CBS) and the Dutch land registry (Kadaster). Metadata are freely downloadable without copyright from the Dutch National Geo Registry: <https://www.nationaalgeoregister.nl/geonetwork/srv/dut/catalog.search#/metadata/effe1ab0-073d-437c-af13-df5c5e07d6cd?tab=general>). The figure is made using statistical software R [R Core Team (2021). R: A language and environment for statistical computing. R Foundation for Statistical Computing, Vienna, Austria. <https://www.R-project.org/>]’.

Due to the reviewers other request on page 4 to focus more on the impact estimates and thus move much other text to the supplements, the map image of figure three has been removed from the main manuscript, and can be found in the supplement S4 (as figure S4.2).

Additional Editor Comments:

In this time, the decisions made by reviewers were split, but I believe that the points both reviewers mentioned can be addressed by appropriate revision. At present the manuscript includes too many kind of methodologies, and as a result, it becomes distracted to some extent. The scope of the manuscript should be clearer through its revision process.

**REPLY:** We thank PLOS ONE for the thorough review. As both reviewers mention that the manuscript includes many methodologies, we followed the advice of both reviewers to move most parts of the scoping review to the supplements and to focus the main document more on the estimated impact sections. As suggested by the reviewers, a very short summary of methods and the main findings of the scoping review now remain in the main text, with reference to all scoping review details in supplements S1-S4. Then, in the manuscript we further elaborated on the methods and results of the impact estimates. This indeed clarifies the scope of the manuscript which is summarized in the abstract as follows: ‘This study describes current knowledge of influenza vaccination in teachers and estimates its potential impact’.

[Note: HTML markup is below. Please do not edit.]

Reviewers' comments:

Reviewer's Responses to Questions

**Comments to the Author**

1. Is the manuscript technically sound, and do the data support the conclusions?

The manuscript must describe a technically sound piece of scientific research with data that supports the conclusions. Experiments must have been conducted rigorously, with appropriate controls, replication, and sample sizes. The conclusions must be drawn appropriately based on the data presented.

Reviewer #1: No

Reviewer #2: Yes

**REPLY:** To better clarify the scientific research we have expanded the description of the methods and results section of the impact estimation (as was also suggested by both reviewers in their separate comments) (page 9-10, lines 144-174 and page 11-19, lines 208-348). We have now also included an additional figure explaining the calculations (figure 2).

 2. Has the statistical analysis been performed appropriately and rigorously?

 Reviewer #1: Yes

Reviewer #2: Yes

**REPLY:** Thank you.

 3. Have the authors made all data underlying the findings in their manuscript fully available?

The [PLOS Data policy](http://www.plosone.org/static/policies.action#sharing) requires authors to make all data underlying the findings described in their manuscript fully available without restriction, with rare exception (please refer to the Data Availability Statement in the manuscript PDF file). The data should be provided as part of the manuscript or its supporting information, or deposited to a public repository. For example, in addition to summary statistics, the data points behind means, medians and variance measures should be available. If there are restrictions on publicly sharing data—e.g. participant privacy or use of data from a third party—those must be specified.

Reviewer #1: Yes

Reviewer #2: Yes

**REPLY:** Thank you.

  4. Is the manuscript presented in an intelligible fashion and written in standard English?

PLOS ONE does not copyedit accepted manuscripts, so the language in submitted articles must be clear, correct, and unambiguous. Any typographical or grammatical errors should be corrected at revision, so please note any specific errors here.

 Reviewer #1: No

Reviewer #2: Yes

**REPLY:** The manuscript has now been edited by a professional English language editor (S. Ebeling, freelance editor who has previously worked for *The New England Journal of Medicine)*

 5. Review Comments to the Author

Please use the space provided to explain your answers to the questions above. You may also include additional comments for the author, including concerns about dual publication, research ethics, or publication ethics. (Please upload your review as an attachment if it exceeds 20,000 characters)

Reviewer #1: This manuscript presents the results of a scoping review and impact assessment of influenza vaccination of teachers in the Netherlands. Given the current discussion around COVID-19 vaccination of teachers and safely opening schools it is a highly relevant research question. Overall the article is clearly written and obviously represents a massive amount of work. However, I think there is too much information in this manuscript for one scientific article. I would recommend that the authors focus on the impact assessment, as the results of the scoping review (namely, there hasn't been much done in this field) are not particularly surprising. I suggest moving the scoping review methods and much of the results to a supplement (or splitting the manuscript into separate articles). I have attempted to provide some detailed comments by manuscript section, but these comments would likely be much more helpful to the authors once there has been a decision on how to proceed in regards to splitting or changing the focus of the current manuscript.

**REPLY:** We appreciate the reviewer’s compliments regarding the relevance of the research question and the amount of work involved. We followed his/her advice to focus more on the impact assessment and to move much of the scoping review methods and results to the supplements (see supplements S1-S4). This is also in line with the same suggestion done by reviewer 2. A brief summary of the scoping review methods and results remain in the main manuscript. And we now elaborated much more on the methods and results section of the impact estimation. We also included an additional figure explaining the impact calculations (figure 2).

Introduction

Page 7 Line 102 - It is not universally true that there is no protection from year to the next. In fact, early life influenza infection has been shown to influenza susceptibility to later life infection This is particularly for H1N1pdm09 which has seen relatively little antigenic drift since it was established as a seasonal virus. Similarly, waning antibody level is another reason for annual vaccination campaigns.

**REPLY:** We agree with the reviewer that this is not universally true. We have removed the section about a lack of year to year protection and we changed the sentence to:

‘With influenza viruses constantly evolving (antigenic drift) [1] and waning antibody levels, a new vaccination campaign is needed annually [5].’ (page 6 lines 86-87)

Be specific about the protection afforded by seasonal influenza vaccines. The 30-50% number reported by CDC is against medically attended, symptomatic infection.

**REPLY:** Thank you for noticing this omission, we have added that this is indeed against medically attended infection:

‘The seasonal vaccine offers partial (roughly 30-50%) protection against medically attended, symptomatic infection if the match between the vaccine viruses and the circulating viruses is strong [6]; it provides less protection if circulating strains deviate from predicted.’ (page 6 lines 87-90)

Page 7 line 117 - this phrasing is a little awkward. I would suggest saying that vaccination is associated with a reduction in absenteeism due to influenza associated illnesses.

**REPLY:** We agree that the phrasing was awkward. We have now rephrased to:

‘Influenza vaccination may also protect employees in sectors other than health care and might reduce absenteeism due to influenza-associated illnesses [9].’ (page 6 lines 103-104)

Methods

The search strategy was clearly described. But why was January 2000 selected as a start date? Is this tied to Dutch vaccine recommendations or other criteria? If there is no rationale for the time restriction I would open it up to include any dates (with the expectation that very few studies were done prior to 2000). Of note, none of the included articles were published before 2007, this tracks with US vaccine recommendations which were expanded in the 2000s and early 2010s.

**REPLY:** The selected period (2000-2020) was not tied to any policy or recommendations. As suggested by the reviewer we have opened up the time restriction to include any dates prior to 2000. This provided two additional scientific papers which after screening were not relevant (they were not about influenza vaccination in teachers). We adjusted the selected search period in the methods section (Page 8, S1, S2) and we adjusted figure 2 to reflect this search strategy change.

Results

Line 220 - Include reasons why scientific articles and grey publications were excluded - are the detailed descriptions of the 12 or the 15

**REPLY:** We agree with the reviewer that we had omitted this information. We have added the following text to the methods of the scientific and grey literature search:

‘Publications were excluded if they were not about influenza vaccination or not about teachers’. (supplement S1, page 1 lines 20-21, and supplement S2, page 1 lines 25-26)

Line 367 - You say absenteeism was reduced by too little to justify continuing the program according to a newspaper article. Was the actual reduction noted?

**REPLY:** Unfortunately the newspaper item did not provide the actual reduction, which we now added to the sentence as follows: ‘The latter stopped offering teacher vaccination as absenteeism reduced too little. However, details on the magnitude of reduction and how and where vaccination was offered by these boards are not given.’ (supplement S4 page 2 lines 77-79)

How was tone of the article (e.g. positive, indifferent) determined?

**REPLY:** We have extended the methods with the following details about how the newspaper sentiment was determined:

‘Positive sentiment was noted when a positive opinion was expressed, or when the article described actual implementation of vaccination of school teachers. A negative opinion or non-implementation were considered as a negative sentiment. When articles did not have a clear overall sentiment because a combination of positive and negative opinions was expressed in the article, we considered the sentiment “multiple”.’ (supplement S4, page 1, lines 32-36)

Line 387 - "we adjusted this number" - How was this adjustment done? What assumptions were made?

**REPLY:** This information was indeed missing. We have now added the explanation to the methods section as follows:

‘Because influenza sick leave and influenza-like illness can be caused by respiratory pathogens other than influenza, we further adjusted these rates by multiplying them by the proportion of specimens testing positive for influenza from sampled patients with influenza-like illness who visited a GP [32-34]. This proportion was from the total Dutch population, as teacher-specific data was not available.’ (Page 9, lines 158-162)

Discussion

Much of the first paragraphs in the discussion are repetition of what was presented in the scoping review section of the results.

**REPLY:** We certainly agree with the reviewer. The scoping review results have now been shortened to a very brief summary with all further details placed in supplements (S1-S4), as was suggested by both reviewers. This removes any repetition that was present in the body text. The main points now only remain discussed and placed into perspective in the discussion section.

Estimates of cases averted and absenteeism have been in the US, but don't seem to be mentioned in the discussion.

**REPLY:** These estimates are indeed available for the USA, albeit not specifically for teacher populations, and should indeed be mentioned. We have now added a reference to estimates in USA health care workers by referring to a review paper that reviewed multiple papers on this topic:

‘Also, a review study of health care workers concluded that the incidence of absenteeism due to influenza-like illness was reduced and its duration shortened in those vaccinated, but an estimate of the magnitude of reduction was unavailable [58].’ (Page 22, lines 422-425)

Overall

In my opinion, the scientific value of this paper is really in the impact assessment. I would reframe the narrative throughout to focus on that, minimizing the scoping review. This would mean a very brief summary of the scoping review methods and results, with most of the detail moved to the supplementary materials. In contrast I think much more detail is needed in the methods section for the impact assessment. Similarly the results section should focus primarily on the impact assessment instead of burying that material at the end after a detailed descriptions of the review.

**REPLY:** We agree with reviewer 2, whose comments are in line with reviewer 1. We adjusted the manuscript to focus mostly on the impact assessment and we moved much of the scoping review methods and results to the supplements (see supplements S1-S4). As the reviewer suggests, only a brief summary of the scoping review methods and results now remains in the main manuscript. In the manuscript we now elaborated much more on the methods and results of the impact estimation (page 9-10, lines 144-174 and page 11-19, lines 208-348). We also included an additional figure explaining the impact calculations (figure 2).

Reviewer #2: Review
In this study, the authors conducted a comprehensive review on flu vaccination administered to school teachers and evaluated its requirements and rationale. I consider that this study’s objectives were (at least partially) achieved by using a combination approach of scoping review, interview, and impact estimation. However, this study is very complex because many issues and study methods are handled in the same paper. I believe this paper is worth publishing. However, some attempts to improve the readability are needed.

**REPLY:** We thank the reviewer for their opinion that the paper is worth publishing if readability is improved. We made large adjustments to improve the readability. The scoping review (including the interviews) is now only briefly summarized in the body text (with reference to supplements S1-S4 for details), which greatly reduces the complexity of the manuscript. Additionally we elaborated the methods and results sections of the impact estimation to better clarify that topic (page 9-10, lines 144-174 and page 11-19, lines 208-348).

1. In my opinion, scoping review, interview (qualitative study), newspaper search, and impact estimation (modeling) seem different. Since the study consisted of synthesizing these four different studies, this manuscript looks like a policy document rather than an academic write-up. To clarify the scope of each study part, I suggest that the authors explain the aims and reasons for which this method was selected in every part.
2. In addition, the deductive theory flow in this study is as follows: 1. There is a lack of school teachers in Netherlands –> 2. The flu outbreaks worsen this situation –> 3. vaccine could save this situation –> 4. Currently, vaccination is not satisfactorily performed -> 5. So how can we do it? I consider this flow to be inconsistent in this manuscript in each part, leading to a decrease in readability. Therefore, I believe that being conscious of this flow can help readability.

**REPLY:** We certainly agree that the flow was not clearly explained in the manuscript. We therefore substituted the final paragraph of the introduction by a concise description of the flow:
‘Although Dutch media have recently reported on influenza vaccination of teachers as one way to decrease schoolteacher shortages, a comprehensive overview of literature is lacking. Therefore, we describe the current knowledge of teacher influenza vaccination using very broad input (scientific literature, grey literature, Dutch newspaper reports and information from key informants). Since knowledge of the impact of this vaccination in teacher populations is crucial for decision-making, but is lacking, we then estimated its potential impact.’ (Page 7, lines 116-121). We did not refer to reviewers points 4 and 5 as they were not part of the opinion or the scope of this manuscript. To better clarify this we added a sentence to the introduction:

‘Traditional risk groups targeted for influenza vaccination in many countries include the elderly (60+) and persons with underlying chronic conditions, because of their higher risk of influenza complications. In the Netherlands, this does not include teachers unless they are individually targeted because of fitting one of those traditional risk groups.’ (Page 6, lines 91-94). Also, touching on that topic, we do make a recommendation in the conclusion: ‘Teacher surveys, in-depth cost-effectiveness studies and a pilot at-school influenza vaccination programme could provide critical information about teacher vaccination.’ (Page 25, lines 485-486).

3. Did the author know the low vaccine prevalence in Amsterdam before this survey was performed? If the authors knew this fact before the survey, I think they should mention the low vaccine prevalence in the Introduction section. This is because the low vaccine prevalence could explain the reason for this study and its rationale naturally.

**REPLY:** Teachers are not a target group for influenza vaccination in the Netherlands. We agree that this was not very clearly stated and we have added the following to the introduction:

‘Traditional risk groups targeted for influenza vaccination in many countries include the elderly (60+) and persons with underlying chronic conditions, because of their higher risk of influenza complications. In the Netherlands, this does not include teachers unless they are individually targeted because of fitting one of those traditional risk groups.’ (Page 6, lines 91-94).

An important reason for the study was the media attention for the potential of decreasing teacher shortages by influenza vaccination and one public health physician wondering if teacher vaccination would actually have an impact on teacher absenteeism. As little seemed known about influenza vaccination specifically in the teacher group we decided to perform a scoping review to provide an overview of the current knowledge on this topic and to identify knowledge gaps more accurately than from hearsay. The main knowledge gap that came forward was the lack of knowledge on the potential impact of teacher vaccination. Therefore we complemented the study with an impact estimation. We have now summarized this in the final paragraph of the introduction as follows:

‘Although Dutch media have recently reported on influenza vaccination of teachers as one way to decrease schoolteacher shortages, a comprehensive overview of literature is lacking. Therefore, we describe the current knowledge of teacher influenza vaccination using very broad input (scientific literature, grey literature, Dutch newspaper reports and information from key informants). Since knowledge of the impact of this vaccination in teacher populations is crucial for decision-making, but is lacking, we then estimated its potential impact.’ (Page 7, lines 116-121).

4. In the Introduction section, the authors mentioned “overviewing any aspect vaccinating teachers against influenza,” however, the aim of the scoping review is identifying “gaps” between existing evidence and required evidence to state the specific purpose of scoping. Therefore, the authors should first define the aim of this scoping review. In this study, I understand that this scoping review aimed to collect evidence on the spread of flu vaccinations among school teachers.

**REPLY:** We agree that this final paragraph of the introduction was not clear. In the beginning of the introduction we added the information that in the Netherlands, teachers are not targeted for influenza vaccination. And we have replaced the unclear final paragraph by the following: ‘Although Dutch media have recently reported on influenza vaccination of teachers as one way to decrease schoolteacher shortages, a comprehensive overview of literature is lacking. Therefore, we describe the current knowledge of teacher influenza vaccination using very broad input (scientific literature, grey literature, Dutch newspaper reports and information from key informants). Since knowledge of the impact of this vaccination in teacher populations is crucial for decision-making, but is lacking, we then estimated its potential impact.’ (Page 7, lines 116-121).

5. Regarding this scoping review, did the authors identify regions with inadequate vaccination evidence based on the author’s purpose? This seems to be mentioned in the Conclusion section, although I could not find it in the main text.

**REPLY:** Influenza vaccination for school teachers in not nationally recommended in the Netherlands. We agree that this is not entirely clear from the conclusion section. To clarify this, we have added a sentence in the introduction and we have now emphasized this in the conclusion section as follows (see bold/italic typing):

In the introduction:

‘Traditional risk groups targeted for influenza vaccination in many countries include the elderly (60+) and persons with underlying chronic conditions, because of their higher risk of influenza complications. ***In the Netherlands, this does not include teachers unless they are individually targeted because of fitting one of those traditional risk groups***.’ (Page 6, lines 91-94).

In the conclusion:

However, most of the research was conducted in the USA, where influenza vaccination is already officially recommended for all citizens (excluding infants <6 months) and vaccination uptake is generally higher than in Europe. ***Grey literature showed that a few European countries nationally recommend influenza vaccination for teachers. In the Netherlands, no municipality, except Amsterdam in 2018 and 2019, was found to offer free influenza vaccination to teachers.*** However, multiple Dutch school boards do offer vaccination to their personnel, according to key informants and some newspaper articles. The main motivation for the offer was a reduction in absenteeism, but vaccine uptake levels were unreported. Unfortunately, details on how and where vaccinations were provided were either not known or not given. (page 20, lines 357-365)

6. The review of newspapers did not have an impact on the integrity of this study. Additionally, I do not know the scientific validity of these methods. Therefore, it may be better to remove this part from this study.

**REPLY:** The grey literature did not provide a full overview of the Dutch media attention for teacher influenza vaccination. Therefore we added a search of a Dutch newspaper database. We have removed almost all details on this topic from the manuscript to supplementary S4. This does indeed not impact the integrity of the main manuscript. As the media attention for influenza vaccination as a way to reduce teacher shortages was one of the reasons for this study we feel it best to still provide these details, but in a supplementary.

7. In the impact estimation part, statements that should be written in the Methods section are frequently seen in the Results section (model selection, way of adjustment, etc.). Please consider ameliorating this structure.

**REPLY:** We agree with the reviewer and we therefore moved those sentences to the methods section.

8. The conclusion is unclear. It could be understandable if the objectives of each part of this study are made intelligible.

**REPLY:** We agree that this needed more clarity. To this end several sections were modified: the final paragraph of the introduction (to clarify the objectives), the first paragraph of the methods was shortened and simplified (also to clarify the objectives), and a few small clarifications were added to the final paragraph of the discussion:

In introduction:

***‘Although Dutch media have recently reported on influenza vaccination of teachers as one way to decrease schoolteacher shortages, a comprehensive overview of literature is lacking. Therefore, we describe the current knowledge of teacher influenza vaccination using very broad input (scientific literature, grey literature, Dutch newspaper reports and information from key informants). Since knowledge of the impact of this vaccination in teacher populations is crucial for decision-making, but is lacking, we then estimated its potential impact.’*** (Page 7, lines 116-121).

In methods:

***‘We conducted a scoping review of the considerations for and impact of influenza vaccination of school teachers. We then estimated the potential impact of teacher vaccination in the Netherlands at different scenarios of vaccine uptake for three influenza seasons (2016-2019).’*** (Page 8, lines 123-125)***’***

In Discussion:

‘In conclusion, international scientific literature on influenza vaccination of teachers remains sparse ***and vaccination of this group is not nationally advised in most countries.*** However, Dutch media ***attention*** is growing, sparked by teacher shortages in recent years. The impact of vaccinating teachers has been particularly understudied, although some key experts and media reports have stated that any school board or other entity that is considering offering influenza vaccination to teachers needs to understand the impact of the vaccination. Our estimates showed that vaccinating teachers against influenza might be associated with a substantial decrease of sick leave days due to that viral infection. Teacher surveys, in-depth cost-effectiveness studies and a pilot at-school influenza vaccination programme could provide critical information about teacher vaccination.’ (page 25, lines 478-486)

6. PLOS authors have the option to publish the peer review history of their article ([what does this mean?](https://journals.plos.org/plosone/s/editorial-and-peer-review-process#loc-peer-review-history)). If published, this will include your full peer review and any attached files.

If you choose “no”, your identity will remain anonymous but your review may still be made public.

**Do you want your identity to be public for this peer review?** For information about this choice, including consent withdrawal, please see our [Privacy Policy](https://www.plos.org/privacy-policy).

Reviewer #1: No

Reviewer #2: **Yes:** Yoshiki Kusama

[NOTE: If reviewer comments were submitted as an attachment file, they will be attached to this email and accessible via the submission site. Please log into your account, locate the manuscript record, and check for the action link "View Attachments". If this link does not appear, there are no attachment files.]

While revising your submission, please upload your figure files to the Preflight Analysis and Conversion Engine (PACE) digital diagnostic tool, <https://pacev2.apexcovantage.com/>. PACE helps ensure that figures meet PLOS requirements. To use PACE, you must first register as a user. Registration is free. Then, login and navigate to the UPLOAD tab, where you will find detailed instructions on how to use the tool. If you encounter any issues or have any questions when using PACE, please email PLOS at [figures@plos.org](mailto:figures@plos.org). Please note that Supporting Information files do not need this step.

*In compliance with data protection regulations, you may request that we remove your personal registration details at any time.* [*(Remove my information/details)*](https://www.editorialmanager.com/pone/login.asp?a=r)*. Please contact the publication office if you have any questions.*
