# Supplementary material for: Influenza vaccination of school teachers: A scoping review and an impact estimation
Source: PLoS One. 2022 Aug 11;17(8):e0272332. doi: 10.1371/journal.pone.0272332 (PMC9371289; doi:10.1371/journal.pone.0272332)
Supplement: Attachment — Submitted filename: Response to Reviewers.docx [file pone.0272332.s008.docx]

**Response to reviewers**

Dear PLOS ONE,

Thank you for your queries and for sending us the favourable evaluation of both reviewers. We gladly addressed the minor concerns raised by them as described below. We hope that we have adequately addressed their concerns. Feel free to contact us would there be any remaining queries.

4. Have the authors made all data underlying the findings in their manuscript fully available?

The [PLOS Data policy](http://www.plosone.org/static/policies.action#sharing) requires authors to make all data underlying the findings described in their manuscript fully available without restriction, with rare exception (please refer to the Data Availability Statement in the manuscript PDF file). The data should be provided as part of the manuscript or its supporting information, or deposited to a public repository. For example, in addition to summary statistics, the data points behind means, medians and variance measures should be available. If there are restrictions on publicly sharing data—e.g. participant privacy or use of data from a third party—those must be specified.

Reviewer #2: No

Reviewer #3: Yes

The underlying data is indeed rather spread out across the manuscript. It is all available in multiple tables which can be found in the 5 supplements as follows:

1. Scientific literature: table S1.2
2. Grey literature: table S2.3
3. Interviews: table S3.1
4. Newspaper monitoring: table S4.2
5. Impact estimate: input parameters available in table 1 in the main manuscript and table S5.1, S5.2, and S5.3 in supplements 5.

6. Review Comments to the Author

Reviewer #2: The authors have corrected their manuscript appropriately in accordance with my suggestions. The readability of the manuscript was dramatically improved by these revisions. However, the Discussion section is excessively long compared to the contents of the study. The following sentences are possibly omitted (or written more briefly). Please consider if the sentences should be removed or left.

Page 20, Lines 362–366
The other research…for teachers.
Page 21, Lines 380–384
Some key information…it are unknown.
Page 21, Lines 389–391
Future research…influenza vaccination.
Page 22, Lines 405–412
In 2019,…in the education sector.
Pages 22–23, Lines 421–423
These are relevant…on working days lost.”
Page 23, Lines 432–437
In school children…in older age.

I suggest that following sentences should be moved.
Pages 24–25, Lines 470–475
Our interview…other than nationally.
Page 22, Line 413 (because the sentences are not relevant to the impact estimation)

Thank you. We now shortened the discussion by removing and shortening sentences. And we made some sections clearer by cutting sentences into two. In detail, the following changes were made:

- *“The other research articles mainly assessed teachers’ attitudes towards influenza vaccination and the vaccination uptake in this group. However, most of the research was conducted in the USA, where influenza vaccination is already officially recommended for all citizens (excluding infants <6 months) and vaccination uptake is generally higher than in Europe. Grey literature showed that a few European countries nationally recommend influenza vaccination for teachers.”* **WAS CHANGED TO: “The other 11 papers mainly assessed teachers attitudes and uptake, mostly in the USA Grey literature showed that a few European countries nationally recommend influenza vaccination for teachers.”**
- *“Some key informants viewed financial incentives as unnecessary or undesirable, but these incentives were reported in grey literature to increase vaccination uptake. Rotterdam decided not to offer free vaccination to teachers after consultation with school boards . Although vaccination uptake and its predictors have been studied in health care workers [50, 51], Dutch teachers’ knowledge of influenza vaccination and their attitudes regarding it are unknown.”* **WAS CHANGED TO: “Some key informants viewed financial incentives as unnecessary or undesirable, but these incentives were reported in grey literature to increase vaccination uptake. While vaccination uptake and its predictors have been studied in health care workers [50, 51], Dutch teachers’ knowledge of influenza vaccination and their attitudes regarding it are unknown.”**
- *“Future research should focus on estimating the effect of vaccinating teachers on sick leave, as many key informants expressed the need for understanding the potential impact of teacher influenza vaccination.”* **WAS CHANGED TO: “Many key informants expressed that they needed to know the potential impact of teacher influenza vaccination. Future research should also focus on estimating the effect on sick leave of vaccinating teachers.”**
- *“In 2019, teacher shortages were 3.4%, 2.7% and 2.0% in the large cities of Amsterdam, Rotterdam and The Hague, respectively; in 2020 increases were expected in those cities to 8.4%, 6.6% and 4.7%, respectively [11]. In these three cities, requests have come from either the council’s education department (Amsterdam) or a political party (Rotterdam and The Hague) to set up a vaccination programme for school teachers or to investigate the feasibility of such a plan. While Dutch health care workers [51, 52] are offered free influenza vaccination to protect their vulnerable patients, decreased absenteeism and uninterrupted care are considered additional benefits [53]; this outcome could be similar in the education sector.”* **WAS CHANGED TO: “In 2019, teacher shortages were 3.4%, 2.7% and 2.0% in the large cities of Amsterdam, Rotterdam and The Hague, respectively. In 2020 these numbers were expected to reach 8.4%, 6.6% and 4.7%, respectively [11]. In these three cities, requests have come from either the council’s education department (Amsterdam) or a political party (Rotterdam and The Hague) to set up a vaccination programme for school teachers or to investigate the feasibility of such a plan. While Dutch health care workers [51, 52] are offered free influenza vaccination to protect their vulnerable patients, decreased absenteeism and uninterrupted care are considered additional benefits [53]. This outcome could be similar in the education sector for which potential uptake and predictors are not yet known.”**
- *“These are relevant results, as confirmed in a more recent Cochrane review from Demicheli (2018): “ We are uncertain about the effect of inactivated vaccines on working days lost.”* **WAS CHANGED TO: “These are relevant results, as there is uncertainty about the effect of vaccines on working days lost.”**
- *“In school children, influenza vaccination showed a positive effect on overall influenza morbidity, not only in schools [59] but also in the community [43, 60]. For the 2009 H1N1 influenza pandemic, it was found that vaccinating school children decreased absenteeism of both teachers and students [61]. Although we focused our research on short-term effects of influenza vaccination, it is important to note that a discussion is ongoing about the long-term effects of repeated vaccination at a young age, which might increase risk of influenza in older age [62].”* **WAS CHANGED TO: “In school children, influenza vaccination showed a positive effect on overall influenza morbidity, not only in schools [59] but also in the community [43, 60]. For the 2009 H1N1 influenza pandemic, it was found that vaccinating school children decreased absenteeism of both teachers and students [61].”**
- *“Our interviews found that Rotterdam school boards considered free influenza vaccination for their teachers, and some even implemented it. These school boards did not appear in our newspaper search (see S4), and therefore, it is to be expected that more school boards in the country offer their personnel influenza vaccination. Two foreign news articles (Australia and UK) also illustrate that, although teacher influenza vaccination is neither nationally advised nor offered for free, it is sometimes organized other than nationally [66-69] (see S1).”* **This was completely moved to supplement 3.**
- **The two sentences starting at line 413 are about how our impact estimation compares to the (little) available other research. Therefore we would prefer to leave it in the manuscript:** ‘Although very limited data are available on the impact of influenza vaccination of specifically school teachers and staff on absenteeism, research has been reported on healthy adults in the general population. A Cochrane review by Demicheli 2014 found that in healthy adults, at least 71 people required vaccination to prevent one laboratory-confirmed case of influenza (CI: 64 to 80)[54].’

Reviewer #3: The authors have come up with an interesting way of bringing forth the impact of vaccination on teachers’ attendance in the Netherland. They have provided supporting data from various sources. This makes it worth publishing.
The authors have answered all the questions put forth by the earlier reviewers and made the necessary changes to the manuscript. They have edited the paper to see that the language used is grammatically correct. Also, they have changed the overall design by concentrating on the impact of influenza vaccination on the Netherlands’ teachers. The tables are very informative in understanding the calculations. The Result section provides all details. The supplementary data is in detail with relevant tables. The authors have put a lot of effort into this project and the information available is ample. They have correctly added the information associated with scoping review in the supplementary section.
The authors have put a lot of effort into correcting the language as well as reducing the content. Yet, the current version needs some more language polishing. Especially for better understanding. The most common issue I found was the sentences are too long. Breaking up the sentences will be helpful.
Below are a few examples of long sentences as well as grammatically incorrect one that needs to be rephrased into smaller sentences.
Line 110: some individual companies and organizations encourage their educational staff
111 to be vaccinated. For example, Amsterdam has offered free vaccination to schoolteachers since
112 2018/2019.
Line 112 rephrase
Line 114 “A review protocol was not registered for this study.”: rephrase
Line 203 “hits in dutch newspaper” rephrase
Line 257 “Absenteeism duration is measured yearly in nearly the total Dutch teacher population” : rephrase
Line 391-396: Long sentences
Line 405-407 Break up into two sentences
Line 419 However, at the perhaps earlier disease stage of sick leave for influenza, our results indicate that only 3.5-9.1 vaccinated teachers were needed to prevent 1 day of teacher influenza sick leave. Rewrite the sentence
Line 423 – 426 Too long
Line 428-431 Too long
Line 454 rephrase
Line 475 rephrase

*Thank you. The full manuscript was revised by two of the authors. Long sentences were rewritten to make them more readable. Others were shortened. Most changes follow the reviewers above suggestions which were changed as follows:*

- *“In the absence of national guidance on influenza vaccination in the educational system, some individual companies and organisations encourage their staff to be vaccinated. For example, Amsterdam has offered free vaccination to schoolteachers since 2018/2019.”* **WAS CHANGED TO: “In the absence of national guidance on influenza vaccination in the educational system, some local companies and organisations encourage their staff to be vaccinated. For example, in Amsterdam, the public health service has offered free vaccination to schoolteachers since 2018/2019.”**
- *“A review protocol was not registered for this study.”* **This sentence is indeed redundant and out of context. As this information is available in supplement 6 we removed this sentence.**
- *“hits in Dutch newspapers”* **WAS CHANGED TO “the number of reports in Dutch newspapers”.**
- *“Absenteeism duration is measured yearly in nearly the total Dutch teacher population”* **WAS CHANGED TO: “Absenteeism duration is measured yearly in almost all Dutch teachers”.**
- *“For this, our impact calculations provide an initial estimate of sick leave due to cases of influenza infection that might be averted by vaccinating teacher population; the reduction in sick leave could be considerable with a moderate to high vaccination uptake. Future research should additionally focus on the cost-effectiveness of teacher vaccination both for schools and for public health generally, and it would ideally consider the potential effects of easier accessibility and financial incentives on the uptake.”* **WAS CHANGED TO: “Our impact calculations provide an initial estimate of influenza sick leave that might be averted by vaccinating the teacher population. The reduction in sick leave could be considerable with a moderate to high vaccination uptake. Future research should additionally focus on the cost-effectiveness of teacher vaccination both for schools and for public health generally. Ideally it would also consider the potential effects of easier accessibility and financial incentives on the uptake.”**
- *“In 2019, teacher shortages were 3.4%, 2.7% and 2.0% in the large cities of Amsterdam, Rotterdam and The Hague, respectively; in 2020 increases were expected in those cities to 8.4%, 6.6% and 4.7%, respectively [11]”* **WAS CHANGED TO: “In 2019, teacher shortages were 3.4%, 2.7% and 2.0% in the large cities of Amsterdam, Rotterdam and The Hague, respectively. In 2020 these numbers were expected to reach 8.4%, 6.6% and 4.7%, respectively [11]”**
- *“However, at the perhaps earlier disease stage of sick leave for influenza, our results indicate that only 3.5-9.1 vaccinated teachers were needed to prevent 1 day of teacher influenza sick leave.”* **WAS CHANGED TO:** **“Laboratory testing and physician consultation might occur relatively late in the disease process. At the earlier disease stage of taking sick leave for influenza, our results indicate that only 3.5-9.1 vaccinated teachers were needed to prevent 1 day of teacher influenza sick leave.”**
- *“Our relatively high estimates of potentially averted absenteeism in teachers may be likely, given the conclusion of Nichol et al (1995) that immunization decreased absenteeism from work due to upper respiratory illness by 43% and absenteeism due to all illnesses by 36% in healthy, working adults [56].”* **WAS CHANGED TO: “Our relatively high estimates of potentially averted absenteeism in teachers may be likely, given the conclusion of Nichol et al (1995). They reported that immunization decreased absenteeism from work due to upper respiratory illness by 43% and absenteeism due to all illnesses by 36% in healthy, working adults [56].”**
- *“Also, a review study of health care workers concluded absenteeism due to influenza-like-illness was reduced and its duration shortened in those vaccinated, but an estimate of the magnitude of reduction was unavailable [58].”* **WAS CHANGED TO: “Another review study of health care workers concluded that absenteeism was less frequent and shorter in those vaccinated, but the magnitude of this reduction was unavailable [58].**
- *“As some teachers were eligible for influenza vaccination because of age (60+) or underlying medical conditions, we expect that we have underestimated, rather than overestimated, impact of vaccinating teachers.”* **WAS CHANGED TO: “But, some teachers probably had received vaccination from their GP because of age (60+) or underlying medical conditions (within the general immunization programme). The actually registered sick leave that we used as input parameter would have been higher had they not been vaccinated within the general immunization programme. Therefore, we expect that we have underestimated, rather than overestimated, impact of vaccinating teachers.**
- *“Two foreign news articles (Australia and UK) also illustrate that, although teacher influenza vaccination is neither nationally advised nor offered for free, it is sometimes organized other than nationally [66-69].”* **WAS CHANGED TO: “Two foreign news articles (Australia and UK) also illustrate that, although teacher influenza vaccination is neither advised nor offered for free at a national scale, it is sometimes organized locally [66-69]”. This paragraph was moved to supplement 3.**

7. PLOS authors have the option to publish the peer review history of their article ([what does this mean?](https://journals.plos.org/plosone/s/editorial-and-peer-review-process#loc-peer-review-history)). If published, this will include your full peer review and any attached files.


If you choose “no”, your identity will remain anonymous but your review may still be made public.


**Do you want your identity to be public for this peer review?** For information about this choice, including consent withdrawal, please see our [Privacy Policy](https://www.plos.org/privacy-policy).

Reviewer #2: **Yes:** Yoshiki Kusama

Reviewer #3: No

[NOTE: If reviewer comments were submitted as an attachment file, they will be attached to this email and accessible via the submission site. Please log into your account, locate the manuscript record, and check for the action link "View Attachments". If this link does not appear, there are no attachment files.]

While revising your submission, please upload your figure files to the Preflight Analysis and Conversion Engine (PACE) digital diagnostic tool, <https://pacev2.apexcovantage.com/>. PACE helps ensure that figures meet PLOS requirements. To use PACE, you must first register as a user. Registration is free. Then, login and navigate to the UPLOAD tab, where you will find detailed instructions on how to use the tool. If you encounter any issues or have any questions when using PACE, please email PLOS at [figures@plos.org](mailto:figures@plos.org). Please note that Supporting Information files do not need this step.

*In compliance with data protection regulations, you may request that we remove your personal registration details at any time.* [*(Remove my information/details)*](https://www.editorialmanager.com/pone/login.asp?a=r)*. Please contact the publication office if you have any questions.*
